# Dedicated developmental programing for group-supporting behaviors in eusocial honeybees

**DOI:** 10.1126/sciadv.adp3953

**Published:** 2024-11-01

**Authors:** Vivien Sommer, Jana Seiler, Alina Sturm, Sven Köhnen, Anna Wagner, Christina Blut, Wolfgang Rössler, Stephen F. Goodwin, Bernd Grünewald, Martin Beye

**Affiliations:** ^1^ Institute of Evolutionary Genetics, Heinrich-Heine University, Düsseldorf 40225, Germany.; ^2^ Behavioral Physiology and Sociobiology (Zoology II), Biocenter, University of Würzburg, 97074 Würzburg, Germany.; ^3^ Centre for Neural Circuits and Behaviour, University of Oxford, Oxford OX1 3SR, UK.; ^4^ Honeybee Research Center Oberursel, Polytechnische Gesellschaft, Goethe-University Frankfurt am Main, Karl-von-Frisch-Weg 2, D-61440 Oberursel, Germany.

## Abstract

The evolutionary changes from solitary to eusocial living in vertebrates and invertebrates are associated with the diversification of social interactions and the development of queen and worker castes. Despite strong innate patterns, our understanding of the mechanisms manifesting these sophisticated behaviors is still rudimentary. Here, we show that *doublesex* (*dsx*) manifests group-supporting behaviors in the honeybee (*Apis mellifera*) worker caste. Computer-based individual behavioral tracking of worker bees with biallelic stop mutations in colonies revealed that the *dsx* gene is required for the rate and duration of group-supporting behavior that scales the relationship between bees and their work. General sensorimotor functions remained unaffected. Unexpectedly, unlike in other insects, the *dsx* gene is required for the neuronal wiring of the mushroom body in which the gene is spatially restricted expressed. Together, our study establishes dedicated programming for group-supporting behaviors and provides insight into the connection between development in the neuronal circuitry and behaviors regulating the formation of a eusocial society.

## Teaser

Innate contributions to collectively performed tasks are developmentally programed in worker honeybees.

## INTRODUCTION

The evolutionary transition from solitary to social living in vertebrates and invertebrates led to sophisticated social behaviors. During the past 50 to 150 million years of evolution, sociality in some species became so elaborate that individuals in the group forego reproduction and changed their behavioral performance to embrace collective behavior, while others specialize in reproduction leading to the development of two castes, queen and workers (*1*, *2*). From behavioral activities of hundreds and sometimes ten-thousands of worker individuals, new properties have emerged at the collective level such as shared brood care, warfare, collective thermoregulation, nest building, and farming, which contributed to the spectacular ecological success of the eusocial species (*2*). The collective tasks and functions cannot be performed by any single individual alone. They require a group and inherited behavioral patterns performed by individual workers. However, understanding how these sophisticated and innate behaviors for social organization manifest through genetically encoded developmental programs remains poorly understood.

Substantial progress has been made over the past several decades in elucidating the genetic basis of behavior. Although a vast number of genes are likely needed for the performance of a behavior, from those in the neurons driving the behaviors through to the development and movement of appropriate anatomical structures (*3*–*6*), key developmental genes have been identified that act during development to program the capacity of specific behaviors.

A gene in vertebrates for such developmental behavioral programming is the forkhead-domain transcription factor gene *FoxP2,* which is possibly involved in speech ability in humans and approaching behaviors in mice (*7*, *8*). In the invertebrate *Drosophila melanogaster,* the *doublesex* (*dsx*) and the *fruitless* (*fru*) gene control the development of different anatomical and molecular sex–specific neuronal populations in the central nervous system (CNS) that underlie sex-specific behaviors, such as male courtship and aggression, as well as female receptivity and post-mating behaviors (*9*, *10*). Thus, a major question has been whether such dedicated development programs also exist for behaviors underlying social living.

The evolutionary rise of sociality is associated with an expansion of the behavioral repertoires and a dynamic interplay of social interactions leading to cooperation. Diverse cues likely control this richness of behaviors in a social and context-specific manner. Given the complexity of these social behaviors, an important question in behavioral biology and genetics has been which aspects of the behaviors are genetically specified to establish group-living features. Other questions from the neurobiology and sociobiology field concern the neural circuitry representation and the cognitive requirements for these social behaviors. These later questions also stem from the debate whether the control of social behaviors in groups requires advanced cognitive and elaborate sensory processing abilities compared to species living solitarily (*11*–*13*).

To understand these behaviors at the molecular and cellular levels, we must examine eusocial insects with strong innate but elaborate behavioral patterns that can be genetically manipulated. The honeybee (*Apis mellifera*) is the ideal eusocial insect for these studies as it provides a combination of elaborate and well-described innate behaviors in the worker caste (*14*, *15*), computer-based behavioral tracking in small colonies (*16*), and powerful methods for genetic manipulations (*17*–*19*) to examine underlying mechanisms.

Of the dozens of behaviors the honeybees display, the behaviors of the worker bees at the nurse stage (usually at the age of 7 to 12 days) are best suited for this study as their behaviors are robust, occur frequently, involve more than seven behavioral tasks, and are required to collectively rear the brood (*14*, *15*). For example, the nurse bees walk on the comb, repeatedly inspecting the hexagonally structured cells. The bees then choose to take up and handle food (nectar or pollen) to feed the developing larvae involving hypopharyngeal gland (HPG) secretions or to clean the empty cells on the comb (*14*, *15*, *20*, *21*). The nurse bees also continually share food with other colony members through mouth-to-mouth transfer (trophallaxis) (*20*, *21*), which involves the expandable part of the gut called the “honey stomach” (*22*, *23*). When worker bees get older, their behaviors gradually shift to other behaviors, such as honeycomb construction or foraging outside for nectar and pollen (*14*, *15*). Juvenile hormone, vitellogenin protein, and differential gene expression are thought to play crucial roles in regulating this age-dependent polyethism (*24*–*27*). Genetic variation, experience, and physiological state influence the preference to engage in particular behaviors (*28*–*30*). However, how the capacities for such social behaviors are molecularly and cellularly programmed during caste development (*31*, *32*) is not known.

**Fig. 1.**
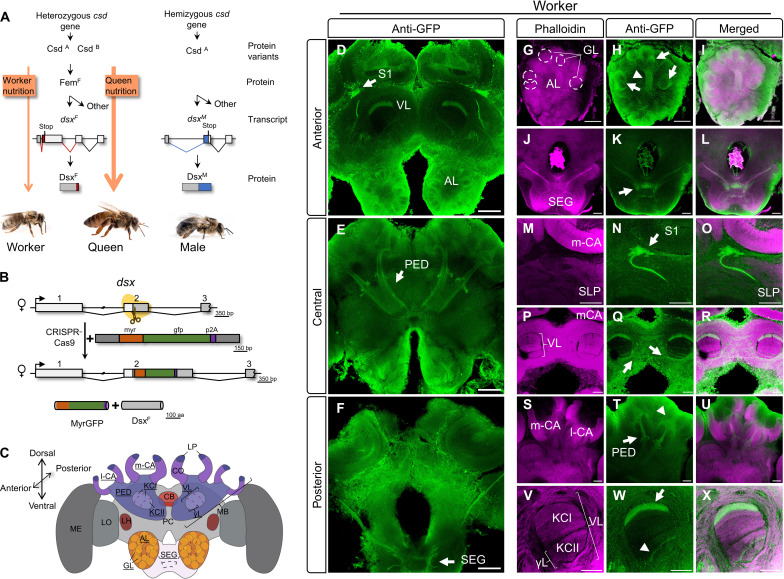
The *dsx^myrGFP^* cells in the brains of worker bees. (**A**) Sex-determination and worker nutrition signals together determine worker characteristics. ^F^/red color, female-specific; ^M^/ blue, male-specific products. (**B**) Targeted insertion of myrGFP. (**C**) Scheme of anatomy of the worker bee brain. MB, mushroom body. LO, lobula. LH, lateral horn. CA, calyx. PED, peduncle. CO, collar. LP, lip. CB, central body. AL, antennal lobe. VL, vertical lobe. γl, γ-lobe. GL, glomerulus. l-CA, lateral calyx. m-CA, medial calyx. KC I/II, projection areas of class I and II KCs in the VL. SEG, subesophageal ganglion. PC, protocerebrum. ME, medulla. Underlined; structures shown below. (**D** to **F**) Overview of *dsx^myrGFP^* expression. Anterior to posterior optical sections through the central brain. Anti-GFP staining. S1, dsx-S1 cluster. (D) 30 μm; (E) 69 μm; (F) 33 μm thickness. (**G** to **X**) Double labeling shown for specific brain areas; anti-GFP (green) and neuropil phalloidin staining (magenta). [(G) to (I)] *dsx^myrGFP^* in OSNs of sensory tract 1 (T1) of antennal nerve (arrowhead) and in olfactory glomeruli (GL) (arrows). Dashed circles; GL. Thickness, 27 μm. [(J) to (L)] Neuronal arborizations labeled in SEG (arrow). Thickness, 33 μm. [(M) to (O)] dsx-S1 soma (arrow) underneath m-CA and l-CA projecting into SLP. Thickness, 18 μm. [(P) to (R)] Commissure (arrows) *dsx^myrGFP^* labeled underneath VL. Thickness, 42 μm. [(S) to (U)] Labeling of class I KCs and projections into PED (arrow). Arrowhead; cell bodies in l-CA and dendritic arborizations. Thickness, 96 μm. [(V) to (X)] Axonal *dsx^myrGFP^*-labeled projections of class I (arrow) in the uppermost layer and class II KCs (arrowhead) in gamma lobe of VL. Thickness, 12 μm. Scale bar, 100 μm [(D) to (F)], 50 μm [(G) to (X)].

In advanced eusocial insect colonies, sophisticated innate behaviors establishing sociality are limited to the worker caste, not to queens. This led to our hypothesis that a dedicated developmental program for social behaviors will be found in the pathway determining the differentiation into the worker castes. Previous work showed that the worker caste is determined by the combined action of the sex determination cascade and a nutrition-derived signal (*19*, *33*–*38*). Different complementary sex determiner (Csd) proteins from heterozygous genotype direct female splicing of the *feminizer* (*fem*) transcripts, which express the active Fem proteins (Fig. 1A and fig. S1) (*33*–*35*). The nutrition signal can only be implemented in females if the Fem protein is expressed given rise to worker ovary characteristics (*19*), suggesting that sex determination and nutrition signal are intertwined to regulate worker caste differentiation. One downstream component the Fem gene regulates is the *dsx* gene. Fem mediates female-specific splicing of the *dsx* transcripts, which then express the female Dsx isoform protein (Dsx^F^; Fig. 1A and fig. S1). Dsx proteins are part of the structurally and functionally conserved *doublesex* and *mab-3*–related transcription factor family (*Dmrt*) and are critical for sex-specific differentiation throughout the animal kingdom (*39*, *40*) and sexual behaviors in insects (*9*, *41*). Dsx^F^ is required for the worker-characteristic differentiation of the worker ovary, suggesting that Dsx^F^ is a component of the worker caste developmental program (*19*). The worker bees do not perform sexual behaviors. However, we found that *dsx* is also expressed in the worker’s brain (*42*) suggesting that the gene may have another role unrelated to sexual reproduction but related to promoting social living behaviors in the worker caste. We, therefore, set out to define the function of the *dsx* gene in specifying worker-specific behaviors, brain organization, and peripheral chemosensory mechanisms.

## RESULTS

### *dsx^F^* is spatially and worker-specifically expressed in the brain

As it is unclear where *Dsx^F^* is expressed in the honeybee brain, we used CRISPR-Cas9–mediated homologous repair to insert myrGFP and the endopeptidase P2A coding sequence into the beginning of the *dsx* coding sequence (Fig. 1B and table S1). The resulting allele, *dsx^myrGFP^*, produces wild-type (wt) *Dsx^F^* and membrane-bound green fluorescent protein (GFP) proteins in the same cell.

In worker bees, which derived from *dsx^myrGFP/+^*-inseminated queens, we detected GFP labeling in distinct brain areas and a selected population of neurons with known behavioral functions (Fig. 1, C to F, and movie S1 to S3) (*43*–*45*). In the antennal lobe (AL), we found that olfactory sensory neurons (OSNs) along sensory tract 1 (T1, arrowhead) of the antennal nerve and in cortical regions of olfactory glomeruli (arrows) were GFP labeled (Fig. 1, G to I) (*46*, *47*). Neurons in the subesophageal ganglion (SEG) were also GFP labeled (arrow, Fig. 1, J to L). We also detected a prominent GFP-labeled cluster with relatively large somata (arrow, which we name dsx-S1) in between and underneath the medial and lateral calyx of the mushroom bodies (MBs) (Fig. 1, M to O). Neurites from this cluster project ventrally and branch out diffusely in the superior lateral protocerebrum (SLP). We found a thin commissure below the vertical lobe (VL) that might be associated with the diffuse arborizations from both dsx-S1 clusters in the SLP on both sides (arrows; Fig. 1, P to R). We found that distinct Kenyon cell (KC) populations were labeled in the MB calyx in which multisensory input is processed and integrated. We found a large bundle of GFP-labeled KC neurites in the peduncle (PED) of the MB (arrow; Fig. 1, S to U) with arborizations in the basal ring and labeling of the inner compact layer of cell bodies of class I KCs in the medial and lateral calyx (arrowhead, m- and l-CA; Fig. 1, S to U). The neurites of this population of KCs proceed along the PED and bifurcate into the medial and vertical lobe of the MB. Strong labeling in the uppermost layer of the vertical lobe shows that most of these neurons are inner compact class I KCs (arrow; Fig. 1, V to X). This group of KCs receives multisensory input in the basal ring of the MB calyx from olfactory and visual projection neurons of the antennal and optic lobes, respectively (*44*, *46*). In addition to this prominent layer in the VL, a small layer of axonal projections from class II (clawed) KCs cells was also GFP labeled in the ventral most part of the VL (γ lobe) (arrowhead; Fig. 1, V to X), which have dendrites spanning over larger regions within all MB calyx subdivisions. These results suggest that within the MBs, the *dsx* gene is specifically expressed in basal ring–associated class I (spiny) KCs and a subset of class II (clawed) KCs.

**Fig. 2.**
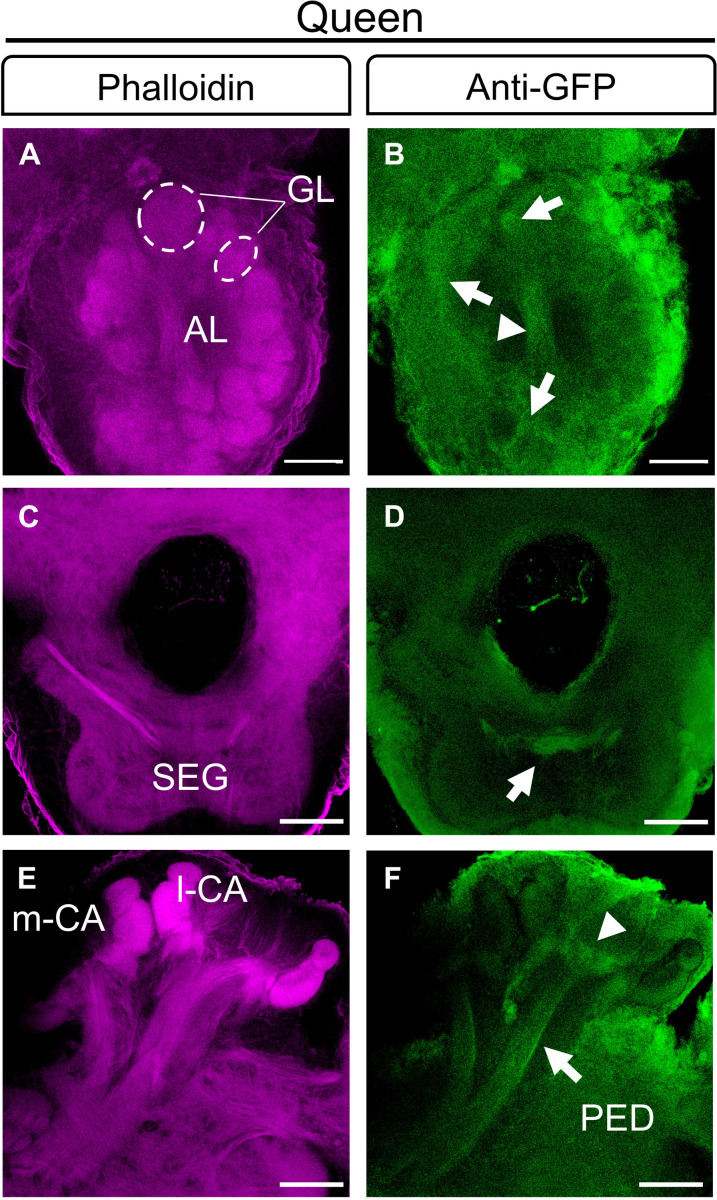
The *dsx^myrGFP^* cells in the brains of bee queens. Double labeling with anti-GFP (green) and neuropil staining with phalloidin (magenta). (**A** and **B**) AL: *dsx^myrGFP^* expression in axonal projections of OSNs in cortical regions of glomeruli (arrows) and sensory tract 1 (T1) of the antennal nerve (arrowhead). Dashed circles in (A) indicate individual olfactory glomeruli labeled with phalloidin. Thickness, 45 μm. (**C** and **D**) Arborizations of *dsx^myrGFP^*-expressing neurons (arrow) in the SEG. Thickness, 48 μm. (**E** and **F**) Neurites from inner compact class I KCs with arborizations in the basal ring (arrowhead) of the calyx and neurites in the PED of the MB (arrow). Scale bar, 50 μm [(A) and (B)], 100 μm [(C) to (F)]. Thickness, 27 μm.

**Fig. 3.**
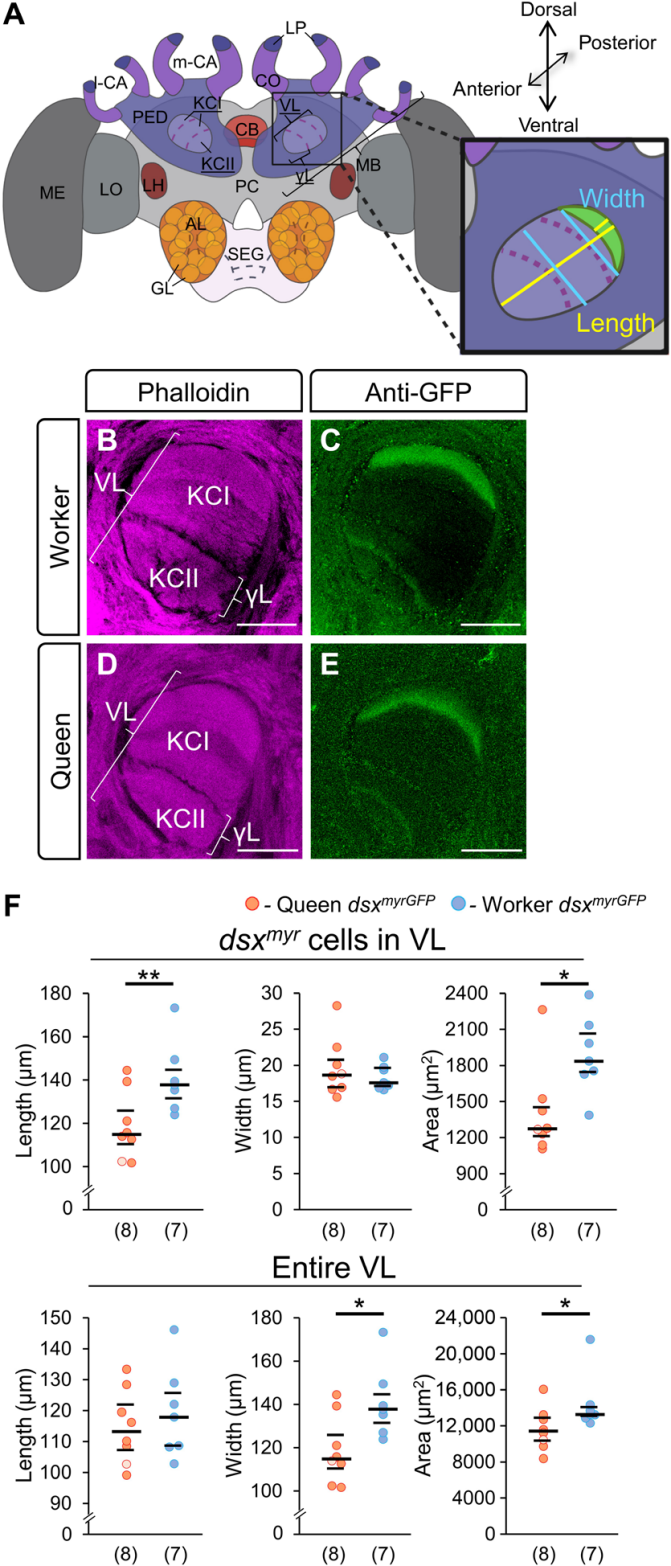
Comparison of *dsx^myrGFP^*-positive neurites in the MB vertical lobes of queen and worker bees. (**A**) Scheme of the brain with inset showing details on quantitative analyses of projection areas in the uppermost (basal ring associated) layer of the vertical lobe (VL) in queens and workers. The length, width, and area of *dsx^myrGFP^*-positive neurites of the KC projections were measured using the position of the largest expansion of the structure as indicated. (**B** to **E**) Examples of *dsx^myrGFP^* expression in the VL of workers and queens. GFP was detected using anti-GFP (green) and the neuropil labeled with phalloidin (magenta). The single queen with the *dsx^myrGFP/myrGFP^* genotype is shown as a dot with a lighter color (details see ”dsx^F^ is spatially and worker-specifically expressed in the brain”). Thickness, 9 and 24 μm. (**F**) Quantitative comparison of the *dsx^myrGFP^*-expressing uppermost (basal ring associated) VL layer and of the entire VL in the brain of queens and workers. The equivalent depth of optical sections was determined by characteristic landmarks in the brain. Length *dsx^myrGFP^*: *P* = 0.008, *z* = 2.66, MWU test. Width *dsx^myrGFP^*: *P* = 0.73, *z* = 0.35, MWU test. Area *dsx^myrGFP^*: *P* = 0.02, *z* = 2.32, MWU test. Length of the entire VL: *P* = 0.58, *z* = 0.58, MWU test. Width VL: *P* = 0.03, *z* = 2.2, MWU test. Area of the entire VL: *P* = 0.04, *z* = 2.08, MWU test. Scale bar, 50 μm [(B) to (E)]. *P < 0.05; **P < 0.01.

We then generated *dsx^myrGFP^* queens by injecting eggs as above and rearing larvae to queens (*18*, *19*). We selected the mutated queens with no mosaicism by deep sequencing of amplicons of the target sites from which we obtained seven *dsx^myrGFP/stop^* and a *dsx^myrGFP/myrGFP^* queens. We compared the pattern of GFP-labeled cells to that of the worker brain. At the gross level, we observed that the queens had similar patterns of GFP-labeled cells as worker bees across optical sections (Fig. 2 and movies S4 and S5). For example, we found the same pattern of axons from OSNS in the T1 tract (arrowheads; Fig. 2, A and B), in the cortical regions of AL glomeruli (arrows; Fig. 2, A and B), in the SEG (Fig. 2, C and D), and in the PED (arrow) with arborizations in the basal ring of the MB calyx (arrowhead; Fig. 2, E and F). However, quantitative examinations of the layer of class I KC axons labeled in the VL suggest that it is larger in worker bees compared to queens (Fig. 3, A to E). The length [Mann-Whitney *U* (MWU) test, *z* = 2.66, *P* = 0.008] and area size (*z* = 2.31, *P* = 0.02) but not the width of KC class I GFP-labeled cells in VL sections were expanded in workers compared to queens (Fig. 3F). The overall length of the VL was not different. In contrast, the overall area size was slightly larger in worker bees (MWU test, *z* = 2.08, *P* = 0.04, Fig. 3F). This expansion in worker bees cannot be explained by the effect of the stop codon in one allele in the *dsx^myrGFP/stop^* queens because the value of the *dsx^myrGFP/myrGFP^* queen did not differ or was even smaller than the values of the *dsx^myrGFP/stop^* queens (one-sample Wilcoxon signed-rank test, length: *z* = 2.03, *P* = 0.04; area: *z* = 0.68, *P* = 0.5; Fig. 3F). Hence, these results suggest a caste-specific dimorphism of *dsx* expressing (*dsx^+^*) cells in the brain. The *dsx-*expressing class I KCs form a larger bundle of axons with arborizations in the VL of workers relative to queens. There may be other caste dimorphic anatomy between workers and queens that we could not detect at this resolution.

We next examined expressions in other tissues of the worker bees using semiquantitative reverse transcription polymerase chain reaction (RT-PCRs). We found that in adult bees *dsx^F^* (Fig. 1A and fig. S1) is consistently transcribed in the brain, ganglia, abdomen, legs, gonads, and fat body, while in the pupae, it is found in the brain, ganglia, thorax, gonads, and fat body (fig. S2). These results suggest that the *dsx* gene in the worker bees is female-specifically regulated, stage-specific, and spatially restricted within the brain and between tissues.

### Brood rearing–related behaviors dysfunction in *dsx^stop/stop^* worker bees

**Fig. 4.**
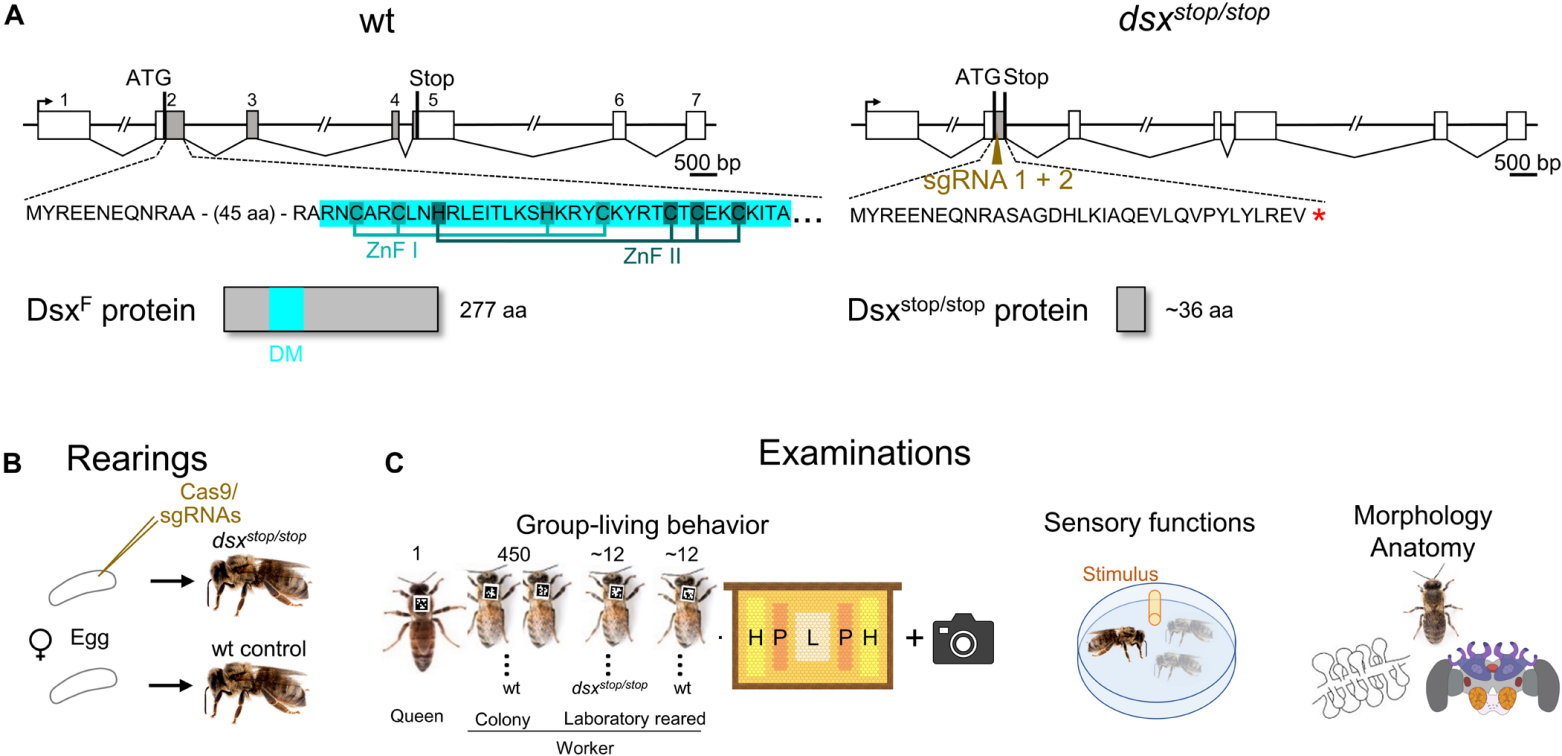
The generation and examination of *dsx^stop/stop^* mutant worker bees. (**A**) Scheme of the genomic organization and coding sequence of the *dsx* gene in wt and *dsx^stop/stop^* bees. Boxes are exons, and interconnected lines indicate female splice variants (male splicing; fig. S1). Gray boxes indicate open reading frame (ORF) with translation starts and stops. The first part of the amino acid (aa) sequence and the resulting protein are presented. ZnF I and II, the two zinc finger motifs of the DM domain (DM, blue box). The brown-colored arrow indicates the target site of the two sgRNAs. (**B**) The generation of the experimental worker bees. Female embryos were reared in the laboratory to the adult stage. Genotyping identified biallelic *dsx^stop/stop^* mutants with no mosaicism. (**C**) The examinations. Left, experimental bees with 450 wt worker bees and a queen were computer-based tracked on a brood comb using 2D barcodes and a camera (*n* = 5 biological replicates). Brood comb had cells with larvae (L), pollen (P), and sugar solution (H). Middle, testing of sensorimotor functions in petri dish assays. Left, morphological/anatomical analyses of mutant bees, including the HPG and the brain.

A key aspect of eusocial living is the collective rearing of the brood. The worker bees at the nurse stage engage into different behavioral tasks to do so. They inspect single cells on the comb, eventually leading to larval feeding, food take up (which we refer to as food handling), or cell-cleaning behaviors, depending on the content of cells (*14*, *15*, *20*, *21*). The nurse bees also share food with other bees (trophallaxis behavior) that is eventually used to feed the larvae. To understand whether the *dsx* gene developmentally specifies aspects of these brood rearing–related behaviors, we generated homozygous *dsx* stop mutants (*dsx^stop/stop^)* in worker bees using the CRISPR-Cas9 method (*19*). We introduced mutations before the essential DNA binding domain, the DM domain, which consists of two intertwined Zinc finger (ZnF) motifs (Fig. 4, A and B, and fig. S1) (*39*). Eggs were injected, and larvae were reared in the laboratory to adults (somatic mutation approach) (*19*). We obtained *n* = 67 (58%) adult *dsx^stop/stop^* worker bees with no mosaicism, which we characterized by deep sequencing of the amplicons of the target site (tables S2 and S3). The survival of *dsx^stop/stop^* and the reared control wt worker bees were not different at the adult stage (*P* > 0.25, df = 1, Fisher’s exact; table S4), suggesting that the biallelic stop mutations did not induce lethality. We examined the behaviors of *dsx^stop/stop^* and wt laboratory-reared control worker bees in small colonies on a brood comb in which distinct areas with the same number of cells contained either larvae, pollen, or sugar solution, while others were left empty. This comb mimics the condition of a brood comb in a colony but with a standardized amount of work to enable behavioral quantification among biological replicates (fig. S3 and table S5). The *dsx^stop/stop^* and wt laboratory-reared worker bees were introduced into a group of 450 wt colony-reared worker bees together with a queen (Fig. 4C). When the bees were 7 to 9 days old, each bee in the group was tracked on the brood comb using individualized two-dimensional (2D) bar codes, which were computer- and video-based recorded (Fig. 4C and table S6) (*16*). The larvae provided in the cells on the comb were reared in all five replicate experiments, suggesting that the nurse bees fulfilled this collective task (table S7).

**Fig. 5.**
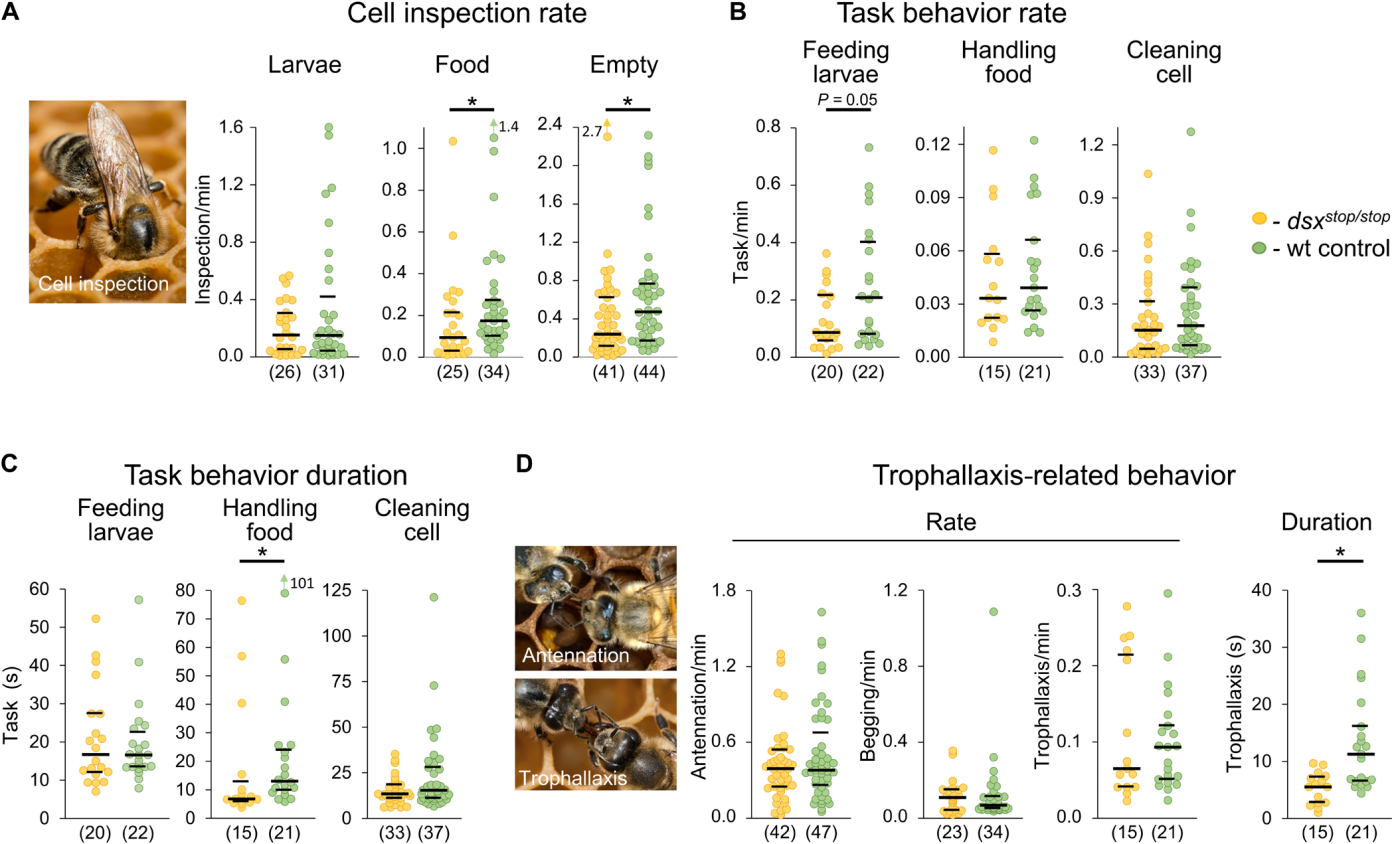
*dsx* gene activity is required for the initiation and sustainment of brood rearing–related behaviors. (**A** to **D**) Comparison of *dsx^stop/stop^* versus wt worker bees. (A) The rate of cell inspections for the different cell types: cells with larvae (*P* = 0.67, *z* = 0.83, MWU test), cells with food (*P* = 0.04, *z* = 2.04, MWU test), or empty cells (*P* = 0.04, *z* = 2.03, MWU test). (B) The rate of feeding the larvae behavior (*P* = 0.05, *z* = 1.93, MWU test), handling of food behavior (*P* = 0.53, *z* = 0.65, MWU test), and cleaning of empty cell behaviors (*P* = 0.29, *z* = 1.05, MWU test). (C) The duration of larval-feeding behaviors (*P* = 0.82, *z* = 0.23 MWU test), handling of food behavior (*P* = 0.02, *z* = 2.28, MWU test), and cleaning of empty cell behavior (*P* = 0.21, *z* = 1.25, MWU test). (D) Rate of antennation (*P* = 0.65, *z* = 0.52, MWU test), begging (*P* = 0.71, *z* = 0.67, MWU test), and trophallaxis behaviors (*P* = 0.95, *z* = 0.06, MWU test). The duration of trophallaxis behavior (*P* = 0.001, *z* = 3.23, MWU test). The median (middle line) and quartiles are presented. *n* values are shown in parentheses. min, minutes; s, seconds. *P < 0.05.

While walking on the comb, worker bees inspect cells by inserting their head into a cell for less than 5 s to detect possible work (*15*). We examined the rates to understand whether the *dsx* gene specifies inspection behaviors. We observed that the rate was markedly reduced by approximately twofold in the *dsx^stop/stop^* versus wt worker bees for the inspection of food-containing cells and cells that were empty (Fig. 5A, table S8, and movies S6 and S7; Mann-Whitney, *z* > 2.02, *P* = 0.04). This result suggests that the initiation of these cell inspections is impaired. However, the inspection rate for cells containing a larva was not reduced. This lack of an effect cannot be explained by the impairment of larval feeding behavior, which could inflate the inspection rate (a portion of inspection behaviors leads to feeding behavior) because the rate of cell inspection and larval feeding behavior together did not differ between *dsx^stop/stop^* and wt bees (fig. S4). Collectively, these results suggest that the *dsx* gene is required for the behavior of inspecting empty and food-containing cells.

After inspecting a cell, a worker bee possibly initiates the behavioral tasks depending on the content of the cell. These task behaviors are larval feeding, food handling, or cleaning of cell. Our observations indicate that the rate at which worker bees performed larval feeding was reduced by a twofold median estimate (Fig. 5B, table S9, and movies S8 and S9; Mann-Whitney, *z* = 1.9, *P* = 0.05). Food handling and cell cleaning behavior was not affected. This result suggests that the initiation of larval feeding behavior was specifically impaired. To determine whether the *dsx* gene specifies the sustainment of these behaviors, we examined the duration of the behavioral task. We observed a twofold reduction in the duration of food handling behavior (from an average of 13.5 s to less than 7 s) in *dsx^stop/stop^* versus wt controls (Fig. 5C and table S10). The other behavioral tasks were not affected, suggesting that the sustainment of food handling behavior was specifically misfunctioned. We did not observe any impairment of the movement patterns during the behaviors (movies S6 to S9), suggesting that the motor programs were unaffected. These results indicate that the *dsx* gene is specifically required to initiate larval feeding and sustain food-handling behavior.

The nurse bees are responsible for consuming and processing pollen to produce easily digestible jelly (*22*). The nurse bees often share this jelly and liquid food from their stomachs with other bees. The transferred food is often used to feed the larvae. Usually, this food sharing requires a sequence of behaviors in which the workers can quit the behavior at decision points or move on in the behavioral sequence. The sequence usually starts with antennation behavior, which is possibly followed by begging behavior and which perhaps results in food-sharing behavior (trophallaxis behavior) (movies S10 to S12) (*22*, *23*). To understand whether the *dsx* gene specifies these behaviors, we examined the rate of begging behaviors and the length of trophallaxis behaviors in the *dsx^stop/stop^* versus wt control bees (movies S13 to S18). We observed that the rates of antennation, begging, and trophallaxis behavior did not differ (Fig. 5D and table S11; Mann-Whitney test, *z* < 0.06, *P* > 0.7). However, the duration of the trophallaxis behavior in *dsx^stop/stop^* versus wt bees was substantially reduced from average 11 to less than 6 s, suggesting a dysfunction (Fig. 5D and table S12; Mann-Whitney, *z* = 3.2, *P* = 0.001;). The movement patterns during the behaviors showed no abnormalities (movies S13 to S18). These results suggest that the *dsx* gene is also required to sustain trophallaxis behavior.

### Movement behavior, maturation, and sensorimotor functions are not affected

**Fig. 6.**
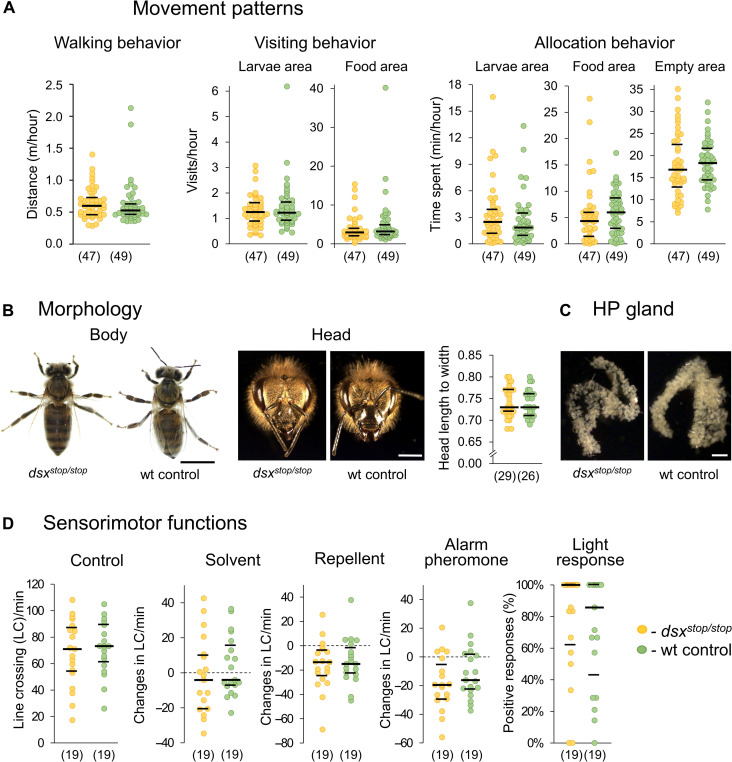
*dsx* activity is not required for movement behaviors, gross morphology, maturation, or gross sensory functions. (**A**) The walking distance of *dsx^stop/stop^* and wt worker bees on the comb (*P* = 0.22, *z* = 1.24, MWU test). The rate of visit behaviors in different comb areas (larvae, *P* = 0.85, *z* = 0.19; food, *P* = 0.17 *z* = 1.36, MWU test). The allocated time to areas (larvae, *P* = 0.33, *z* = 0.97; food, *P* = 0.06, *z* = 1.86; empty, *P* = 0.36, *z* = 0.92, MWU test). (**B**) Example of body (scale bar, 5 mm) and head morphology (scale bar, 1 mm). To the right, head length relative to head width (*P* = 0.87, *z* = 0.16, MWU test). (**C**) Example of the HPGs. (**D**) Sensorimotor function examinations using line crossings (LC). No stimulus (control): *P* = 0.64, *z* = 0.48, MWU test. Changes in LC in response to the solvent isopropanol (*P* = 0.3, *z* = 1.05, MWU), the repellent benzaldehyde (*P* = 0.77, *z* = 0.31, MWU test), and the alarm pheromone IPA (*P* = 0.3, *z* = 1.07, MWU test). The *dsx^stop/stop^* mutant and wt worker both responded to benzaldehyde (*P* < 0.01, *z* > 2.6) and IPA (*P* < 0.03, *z* > 2.25, one-sample Wilcoxon signed-rank test). The responses to light pulse (*P* = 0.67, *z* = 0.48, MWU test). The median (middle line) and quartiles are presented. *n* values are shown in parentheses. min, minutes.

To understand whether the impairments in behavior result from general defects, we examined walking behavior, morphology, and stimulus perception. The walking distance (Fig. 6A and table S13; MWU test, *z* = 1.24, *P* = 0.22) and the visiting behaviors of the comb areas did not differ between the *dsx^stop/stop^* and wt worker bees (Fig. 6A and table S14; MWU test, *z* < 1.37, *P* > 0.17). This result suggests that cell inspection and task behavior impairments cannot be explain by altered movement and visit area patterns on the comb. In addition, the time the *dsx^stop/stop^* bees spent in different areas of the comb was not affected as revealed from wt bee comparison (Fig. 6A and table S15). Possibly, there is a trend of the *dsx^stop/stop^* bees spending less time than the wt bees in the area of the food (MWU test, *z* = 1.86, *P* = 0.06), which may reflect the twofold decline of cell inspections in the food area, which will affect the time spent but not the visit rate. External morphological defects cannot explain the behavioral dysfunctions in *dsx^stop/stop^* bees. The triangular-shaped head morphology, the sex-specific antennal/abdominal segments, and the overall body morphology of the *dsx^stop/stop^* and wt worker bees did not differ upon close and quantitative inspections (Fig. 6B, fig. S5, and table S16; Fisher’s exact test, *P* = 1, df = 1; MWU test, *z* = 0.16, *P* = 0.87).

Bees maturate to the nurse bee stage in the colony usually when they are 7 to 12 days old. To determine whether the *dsx^stop/stop^* worker bees entered the nurse stage, we examined whether the bees developed HPGs and ascini, which produce secretions for the feeding of the larvae (*48*). All the examined *n* = 23 *dsx^stop/stop^* worker bees had developed HPGs similar to those of the wt control worker bees (table S16). The HPGs were composed of acini, densely packed along the collecting ducts (Fig. 6C), suggesting that the *dsx^stop/stop^* mutant bees had entered the nurse stage. Furthermore, as previously reported (*19*), we observed gross malformations of the worker-characteristic reproductive organs that differ from queens (fig. S6 and table S16). The gross malformations were observed in 50% of the *dsx^stop/stop^* mutants, suggesting that the expressivity of the mutant phenotype varies (fig. S6 and table S16).

To understand whether gross motor and sensory functions were compromised, we examined response to different odors and light (Fig. 6D and fig. S7), we demonstrated that loss of *dsx* function mutation did not affect the response to the repellent benzaldehyde (*49*) (MWU test, *z* = 0.31, *P* = 0.77), the alarm pheromone component, isopentyl acetate (IPA) [(*50*); MWU test, *z* = 1.07, *P* = 0.3], or light (MWU test, *z* = 0.48, *P* = 0.67). As a control, we showed that the *dsx^stop/stop^* worker bees behaviorally responded to the stimulus benzaldehyde and alarm pheromone (test against zero, one-sample Wilcoxon signed-rank test, benzaldehyde: *z* > 2.6, *P* < 0.01 and IPA: *z* > 2.25, *P <* 0.03). These quantitative results indicate that gross motor and sensory functions were not affected in *dsx^stop/stop^* worker bees. These results suggest that general abilities of stimuli perception and walking behaviors were intact in the *dsx^stop/stop^* worker bees. We conclude that beside the development of the usually sterile reproductive organ, the *dsx* gene is specifically required to initiate and sustain specific worker behaviors.

### Developmental organization of the brain’s central integrative center is impaired

**Fig. 7.**
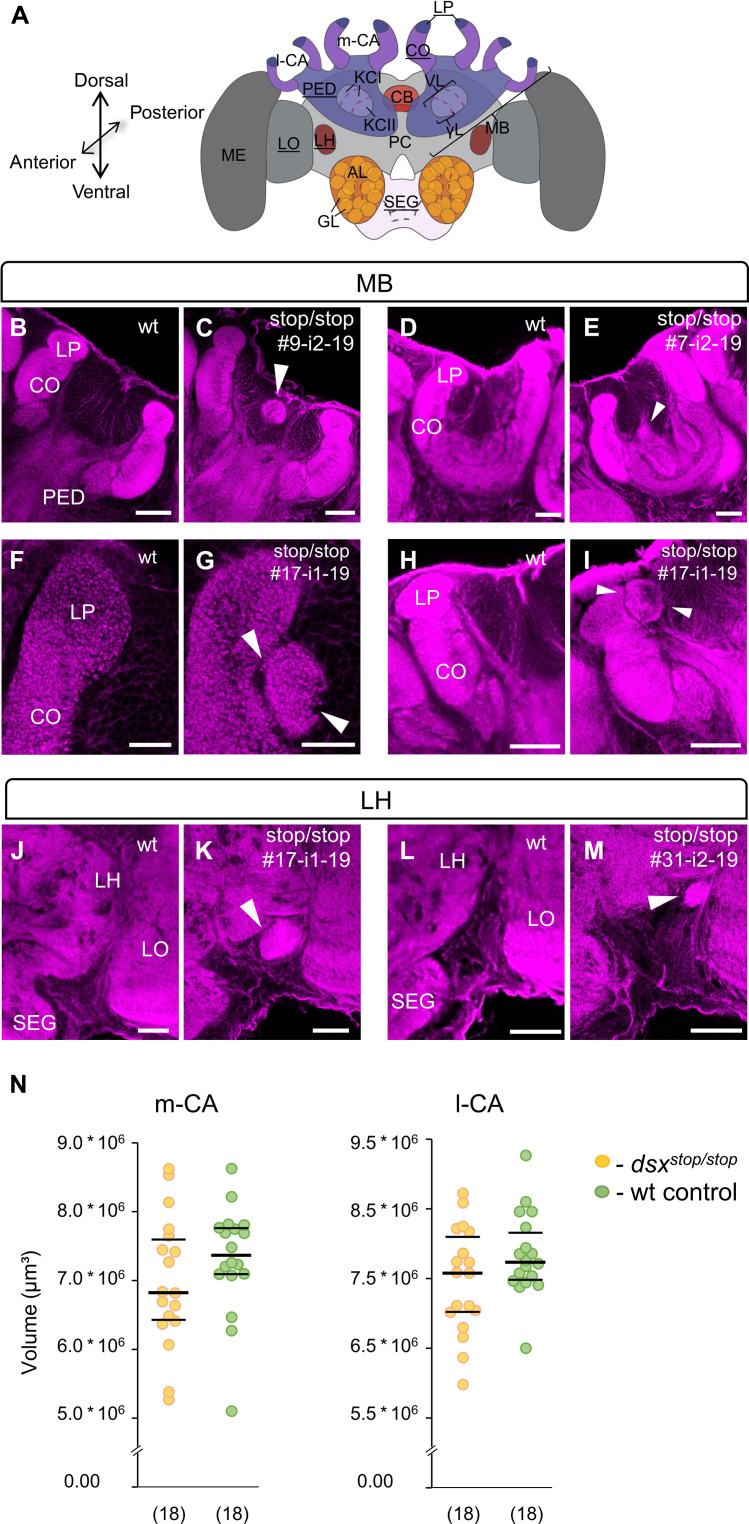
The *dsx* gene is required for proper differentiation of the MB and LH area. (**A**) Scheme of the anatomy of the worker bee brain. Underlined labels highlight the structures shown below. (**B** to **I**) Single optical sections of wt and different *dsx^stop/stop^* worker bees in the MB area. The brain tissue was stained using phalloidin. Abbreviations in labels are indicated in (A). Arrowheads mark additional structures or malformations. Further explanantions in the text. (**J** to **M**) Single optical sections of wt and two *dsx^stop/stop^* worker bee brains in the LH area. *dsx^stop/stop^* worker bees were independently mutated. The brain tissue was stained using phalloidin. Abbreviations in labels are indicated in (A). Arrowheads mark additional neuropil structures adjacent to the LH. Scale bars, 50 μm. (**N**) Volume measures of the CAs. MWU test, medial CA, *z* = 1.28, *P* = 0.2; lateral CA, *z* = 1.25, *P* = 0.21.

We next asked whether the *dsx* gene is a developmental regulator that specifies the anatomy of the worker’s brain. We examined the anatomy of 26 *dsx^stop/stop^* worker bee brains. We used f-actin staining with phalloidin to highlight synaptic neuropils and examined stacks of confocal images of worker bee brains. We found that 23% of the 26 mutants had malformations at a gross observation level. This observation is significantly different to the 29 laboratory-reared wt worker bees that never had such malformations (*P* < 0.01, df = 1, Fisher’s exact test; table S17 and Fig. 7). We repeatedly observed (*n* = 5) spheroidal neuropil-like structures preferentially located on the inside of the MB calyx cup that is typically occupied by cell bodies of KCs (arrowheads in Fig. 7, B to I) together with general malformations of the MB calyx (Fig. 7, D and E; table S18; and movies S1 and S19 to S22). The example in Fig. 7G shows that these neuropilar abnormalities contain microglomerular structures, which comprise clusters of synaptic neuropils at atypical locations. In *n* = 2 individuals, we observed additional structures of very dense neuropil in the lateral horn area (arrowheads in Fig. 7, J to M; table S18; and movies S1, S19, and S23). These results suggest that the *dsx* gene is required for the developmental organization of neuropils and wirings in the MB area. We examined whether these malformations increase the dysfunction of the behaviors. However, the behaviors of these MB malformed workers were in the range of the other mutant workers (fig. S8). Because the expressivity of *dsx* mutant phenotype varies greatly from strong to mild (table S16) (*19*, *51*), we next examined the volume of the calyces (CA) in the other *dsx^stop/stop^* worker bee with no gross MB malformations to find more subtle changes. We found a tendency that the volume of the CAs were slightly smaller relative to wt (Fig. 7N, MWU test, medial CA: *z* = 1.28, *P* = 0.2; lateral CA: *z* = 1.25, *P* = 0.21). There may be other subtle changes that we cannot detect at this level of analysis, which is limited by a lack of resolution at the cellular level.

The brood rearing–related behaviors on the comb are primarily controlled by the sensation of chemical cues from the local environment. Chemosensory receptors are used to perceive molecules from the environment. They are expressed in the OSNs of antennal sensilla, for which we found *dsx* expression in the sensory axons within the AL (Fig. 1, G to I). To understand whether the transcription factor gene *dsx* specifies mechanisms of olfactory and/or gustatory reception, we examined the expression of odorant receptor (OR), odorant-binding protein (OBP), gustatory receptor (GR), and chemosensory protein (CSP) genes in the antennae of mutant bees. We observed that none of the OR, OBP, GR, CSP genes or other genes (*n* = 9361) were differentially transcribed between *dsx^stop/stop^* and wt worker bees, as revealed by RNA sequencing (RNA-seq) analysis (fig. S9). These results suggest that *dsx* does not control the transcription of chemosensory receptor–encoding genes. We conclude that the *dsx* gene is not used to specify worker behaviors at the level of the chemosensory receptor genes.

## DISCUSSION

### The *dsx* gene acts as a developmental regulator to specify worker behaviors

The behavioral patterns that build the basis of eusociality are usually neither taught by adult individuals nor are they learned by the young. They are inherited—programed into the development of the individual by genes that elicit specific behavioral patterns in the adults. Because eusocial behavior is inherited, there must be genes whose products govern molecular and cellular mechanisms as well as neuronal circuitry properties that innately program behavior of individuals in such a way that coordinated behavior of a colony acting as a single organism emerges. However, it was unknown whether there are dedicated developmental programing that specifies the worker-specific innate behaviors into adult bees. By introducing biallelic frameshift mutations into worker bees leading to translation stops, we showed that the activity of the *dsx* gene is necessary for inspection, brood rearing, food handling, and food exchange behaviors. These activities and task behaviors are not displayed by the queen caste and the males. They contribute to the collective brood rearing at the group level. Whereas these behaviors were specifically impaired, the general sensorimotor functions of the worker bees were unaffected, suggesting that the *dsx* gene dedicatedly programs the identity of the worker-specific behaviors into the worker caste. Previous work identified molecular determinants that act during the shift from inside to outside the nest behaviors. For example, juvenile hormone and vitellogenin play crucial roles in the shift in this age-dependent polyethism (*24*–*27*). Other data focused on behavioral states and the transcriptome patterns in the brain (*6*, *27*, *52*). For example, the authors found massive transcriptionally differences of 5839 genes between in-hive and foraging bees (*27*). Other types of data rely on the use of genetic variation to study social behaviors (*30*, *53*) and identified by quantitative trait mapping an inherited basis and genomic loci harboring multiple genes. However, in no case that there has a developmental gene been identified that specifies the identity of these worker-specific behaviors into the worker caste. The rate and duration of task and task-related behaviors specified contribute to collective brood-rearing work, which defines a eusocial society. Hence, our result suggest that we have characterized a key developmental regulator for the programing of social worker behaviors.

We further showed that the *dsx* gene’s activity (*19*) acts as a developmental regulator in the worker bee’s brain. Dsx^F^ thereby controls the proper developmental organization of the MB, with *dsx* mutants showing extra neuropil structures and malformations of the MB calyx neuropil in a proportion of mutants, suggesting that the *dsx* gene is involved in the developmental guidance of neurons in the MB calyx. Because other *dsx* mutant phenotypes show varying expressivity (table S16) (*19*, *51*), more subtle effects might be present in the MB, but detecting those may require higher resolution at the cellular level.

The sex-specific activities of the *dsx* orthologs are in invertebrates and vertebrates integral for sexual differentiation (*19*, *39*, *40*). There is also evidence in other insect species that the *dsx* gene shows plasticity in its regulation of traits depending on internal nutritional states (e.g., horn and mandible formation in the dung and stag beetles, respectively) (*54*, *55*). However, in honeybees, *dsx* is part of the caste determination. Dsx^F^ together with the nutrition signal regulate the caste-specific development of the worker and possibly the queen ovary (*19*). Hence, a key question is how the *dsx*-dependent identity of the worker-specific behaviors is possibly realized in the nervous system. Here, we demonstrate that Dsx^F^-expressing neurons form anatomic differences in workers versus queens. The dsx^+^ basal ring–specific class I KCs in the MBs are caste dimorphic. This result suggests that worker bees have larger neuronal projections in the VL with more connecting dsx^+^ KC class I neurons than queens. Hence, our results demonstrate that *dsx* is a developmental regulator of the MB and is involved in establishing worker-specific anatomic identity in class I KCs of eusocial honeybees.

### Selection and duration of behaviors are developmentally scalable features

Organisms living socially have a rich behavioral repertoire that contributes to different tasks in and outside the colony. Their control is mediated by stimuli that are precepted from the environment and social partners. Hence, an important question in behavioral biology and genetics is about which aspects of these behavioral capacities are genetically specified that establish the features for social living. Here, we show that specifically the rate and duration aspects of the behaviors, but not the movement patterns, are innately programed. The mutants approximately show a 50% reduction in the rate and duration of task and task-related behaviors. Because the collective tasks require many bees and repeated behavioral performances of a worker (for example, 52 cell inspections and 13 larval feedings are performed on average by a single worker bee per hour in our experimental setting), a 50% reduction in each worker is a substantial effect on the collective outcome and social organization. This further supports the notion that the *dsx* gene is a key developmental gene for the programming of group-supporting behaviors in the worker caste. Specifically, we showed that *dsx* specifies the rate a worker bee inspects a cell and finds work during the bees’ walks on the comb (*15*, *56*). We also found evidence that *dsx* specifies the rate a worker bee feeds larvae with a mixture of protein-enriched pharyngeal gland secrets, pollen, and nectar (*21*,*22*), a behavioral capacity representing a hallmark of eusocial organization (*1*, *2*). All these aspects were specified for specific cues the worker bee encountered in the larval, food, or empty cells, suggesting that behavioral specifications are context specific.

Our results also suggest that the duration of food-related behaviors is specified, suggesting another level of innate control. We showed that the *dsx* gene specifies the duration of the food-handling behaviors in the cells (*15*) and the food exchange behaviors among colony members (*22*, *23*). How is the duration of the behavioral performances possibly specified? We suggest that sensory feedback control for the behaviors that are developmentally specified must exist. The perception of cues during the behaviors will control whether the behavior will be further performed, making the performances less rigid and adjustable to local demand (*57*).

Most of the work in the colony cannot be performed by a single bee alone. The workload in the colony requires small and repeated contributions from many bees that collectively fulfill the work. This raises the crucial question how a developmental program can specify behavioral capacities in individual bees in such a way that behaviors in a colony acting as a functional unit emerges. Previous work showed that single bees differ in their experience and genotype make up through genetic variation that determines which bee engage in a behavioral task for a given stimulus [response threshold (RT) model]. A bee below this threshold will behavioral respond to the task stimulus, while a bee above the threshold will not respond. This variation in RTs regulates the fraction of bees in the group that perform this task (*28*, *29*, *53*). Our results now showed that *dsx* developmentally specifies not all but the rate and duration of behavioral performances at some scale for a given stimulus and standard conditions used. Hence, the rate and duration aspects of task behaviors are developmentally programed at some scale. This establishes a programable mechanism for social living because the innate rate and/or duration of performing behavior quantitatively determines the strength of the social relationship the bees have with other bees and the work they share together. For example, the innate rate of brood feeding will specify how often a bee will care for the brood, while the innate duration of the trophallaxis will specify how long the food is transferred between social partners. Even small differences in this scalability will have profound consequences at the collective level because the repeated task engagements and the many worker bees involved amplify the effect. For example, even small changes of the innate scale of longer trophallaxis behaviors (in our experiment, six trophallaxis behaviors were performed per worker per hour) will alter the amount of liquid food circulating in the colony. How is the scalability of the behavioral performance possibly specified? We propose that the *dsx* gene may specify the intensity of neuron activation, the type of inhibitory and stimulatory regulation in a neuronal network, and/or the number of activated neurons (*58*) for stimulus processing leading to motor program control. Hence, our results show that selection and duration of the behaviors are innate, context dependent, and scaled by a dedicated developmental program uncovering mechanisms of innate specifications of eusocial living behaviors.

### *dsx* gene operates in brain areas that integrate and evaluate sensory information

How the behavioral control in eusocial societies is possibly represented at the level of neural circuitry is still rudimentary. Our reporter gene studies now showed that Dsx proteins operate in spatially highly restricted areas of the worker bee brain and at different sensory information processing and evaluation levels. This expression includes OSNs that project from the antennae to glomeruli in the AL and express OR proteins (*45*, *59*, *60*). The transcription factor gene, *dsx,* does not regulate the expression of the chemosensory receptor proteins, which excludes peripheral chemosensory mechanisms as a possible source for the specification of worker behaviors. However, *dsx* may still affect olfactory processing at the level of AL glomeruli. *dsx* may act on selected neurons in the SEG and influence information processing from other sensory systems on the mouthparts, i.e., gustatory and mechanosensory input (*45*, *59*). Most prominently, *dsx* operates developmentally in selected groups of KCs, the projections of which show caste-dimorphic differences in the VL. Most are class I (spiny) KCs of the inner compact type associated with the MB calyx basal ring. The basal ring receives multisensory input from both olfactory and visual modalities, and KCs in the basal ring may even integrate both modalities that are mapped in close vicinity. These findings suggest a potential role of *dsx* in specifying the neural circuits underlying multisensory information processing affecting behavioral decisions (*43*, *44*, *57*). A minor population of class II (clawed) KCs also expressed *dsx*. A notable feature of class II KCs is that their claw-like dendritic arborizations span over larger regions of all regions of the MB calyx, the olfactory (lip), visual (collar), and basal ring, indicating that these KCs also integrate different sensory modalities. Thus, *dsx*’s role in specifying innate behaviors may be associated with *dsx*^+^ neurons involved in processing of multisensory information. One possible mechanism of specification is the presence of a larger population of activated class I KCs in the worker bee (as the caste dimorphism suggests) that can activate a higher number of MB output neurons to activate a motor program. The *dsx* expression in the fat body may also indirectly affect the function of the brain and behavioral control, as shown for the fruitless protein that is secreted from the male fat body and influences the CNS and the courtship song in *D. melanogaster* (*61*).

There is a long-standing debate about whether organisms living socially require higher cognitive and/or larger neuronal processing abilities than those living solitarily (*11*–*13*). The *dsx’s* role in specifying behaviors in socially living worker bees and solitarily living *D. melanogaster* establishes a rare opportunity to evaluate this hypothesis by comparing the gene’s function and expression in the brain of the two species. As in the honeybee, the *dsx* transcripts undergo in *D. melanogaster* sex-specific alternative splicing to encode either a male- or female-specific isoform (*62*). In *D. melanogaster*, *dsx* expression is highly regulated in both male and female flies, as shown by its temporally and spatially restricted expression patterns through development, with only a select group of neurons expressing *dsx* (*9*, *63*, *64*). A detailed analysis of *dsx* expression in both the male and female CNS found that *dsx*-expressing neuron clusters are sexually dimorphic in cell number and connectivity—None of the *dsx*^+^ neuron clusters are sexually monomorphic (*64*). Many of these *dsx* higher-order neurons in the brain act as key sex-specific processing nodes of sensory information that are essential for the execution of sexual behaviors [reviewed in (*65*)]. However, unlike in *D. melanogaster* [and *Bombyx mori* (*66*)], *dsx* in honeybees also operates in KCs, suggesting a not yet described function and expansion to the MBs. This high-order center integrates sensory information and evaluates them for decision-making by comparing incoming sensory input with stored information that has been acquired via sensory integration, learning, and memory formation (*43*, *44*, *57*, *67*). Besides MB’s historically defined role in learning, innate behavioral decisions and olfactory learning were recently shown in *D. melanogaster* to share circuitry of the MB (*68*, *69*). This suggests the potential that the prominent *dsx*^+^ cells of the MB are possibly involved in the innate decision process that were impaired in the *dsx* mutants. The robust behavioral effects together with a selected population of labeled neurons now offer an opportunity to examine the representation and mechanisms of the *dsx*-dependent behavioral controls at the level of neural circuitry.

### The *dsx*’s role in specifying worker behaviors were co-opted from sexual behavior

It is largely unknown how innate behaviors required for social and eusocial organizations genetically evolve through developmental programs. Previous data informed us about the molecular rate, genome evolution, gene family expansions, signatures of selection, and changes in gene regulatory network at the genome scale (*70*–*72*) that are associated with the origin of sociality or eusociality.

In the solitary living species, the fruit fly *D. melanogaster* and the wasp *Nasonia vitripennis* (a hymenopteran sister group of the honeybee), the *dsx* gene is involved in specifying sexual behaviors (*9*, *41*, *63*, *73*). Parsimony evolutionary inference from the two species and the honeybee suggests that the *dsx* gene evolutionarily gained a new role in the honeybee lineage to specify social living behaviors in the worker bees. This new function has been evolutionary co-opted from its ancestral function, the specification of sexual behaviors. We conclude that co-opting a gene from its sexual to a social living–specifying function is an evolutionary path and mechanism involved in the origin of eusociality, which links with the gain of developmental control in the high-order center, the MB.

## MATERIALS AND METHODS

### Honeybee handling procedures

The honeybees were collected from *Apis mellifera carnica* colonies (western honeybee) at the bee yard of the Heinrich-Heine University, Düsseldorf, Germany. Female eggs were collected from naturally mated queens, which were maintained in small nuc colonies with five combs (Holtermann, Germany). To collect female eggs, the queens were caged in Jenter egg-collecting cages (Jenter queen rearing kit, Karl Jenter GmbH, Frickenhausen, Germany). For the tracking, we collected newly eclosed bees (0 to 24 hours old) from a brood comb that was maintained in an incubator at 34°C. The laboratory rearing of worker bees was done as previously reported (*19*). We grafted the newly hatched larvae into plastic cups (#4963, Heinrich Holtermann KG, Brockel, Germany) with worker diet # 7 (53% royal jelly, 4% glucose, 8% fructose, 1% yeast extract, and 34% autoclaved water) (*74*). To obtain bees with fully developed worker characteristics, we experimentally determined the amount of food provided, which was 170 μl per larvae. The larvae were kept at 34°C and 90% relative humidity. The latter was generated using saturated solution of K_2_SO_4_ (*75*). Before defecation, the larvae were transferred onto Kimwipe papers (Delicate Task Wipers, #066664, Kimberly Clark) and were kept in petri dishes for 2 days at 70% relative humidity, which we generated using saturated NaCl_2_ solutions (*75*). After defecation, the prepupae were separated into plates with 24 wells (#92424, Peter Oehmen GmBH, Essen, Germany) in which filter papers were placed (15 mm, grade 413; VWR, International GmbH, Darmstadt, Germany). Once they started walking, they were marked and maintained in small cages together with eclosed wt workers coming from colonies (#20104; Imkereifachhandel Jasniak, Trossin, Germany) in which water and sugar paste supplemented with pollen (#7032; Heinrich Holtermann KG, Brockel, Germany) was provided. To obtain myrGFP-mutated worker or queen bees, we reared the queens as described (*17*, *76*). To obtain myrGFP worker bees, 12- to 19-day-old myrGFP queens were inseminated with wt drones using standard insemination techniques. The queens were treated with CO_2_ 1 day before insemination. Inseminated queens were maintained in small nucs (“Kieler Begattungskasten,” Holtermann, Germany) with wt worker bees. The nucs were kept in a containment so that mutated animals were not able to escape into nature. To obtain newly eclosed *dsx^myrGFP/+^* worker bees, combs with capped cells were maintained in an incubator at 34°C.

### Genetic manipulation procedures

Eggs were collected every 1.5 hours using the Jenter egg collecting system (*17*). To induce stop codons, single-guide RNA 1 (sgRNA1) and sgRNA2 (table S1) were injected together with Cas9 protein (New England Biolabs, Ipswich, MA), which target base pair (bp) position 31 (sgRNA1) and 201 (sgRNA2) downstream of the start codon (fig. S10). Four hundred picoliters of the sgRNA/Cas9 mix was injected per egg (*19*) using Cas9 protein (375 ng/μl) and sgRNA1 and sgRNA2 at equal molar ratio. Needles were custom made (Hilgenberg, Malsfeld, Germany) as described in (*17*). The sgRNAs induced deletions of approximately 170 bp or mediated indels (sgRNA2), which both frequently produced frameshifts of the open reading frame and this before the essential DM domain of the *dsx* gene (*19*). Bioinformatic predictions suggest no off targets for these sgRNAs (*19*). Furthermore, the *dsx^stop/stop^* mutation were independently induced in each worker bee (thus representing independently mutated bees) making an off-target effect, which may also be induced at some rate, as an explanation for the phenotype effect unlikely. To introduce myrGFP coding sequence, sgRNA1 together with DNA donor fragment were injected (table S18) following previously described procedures (*18*). The injected volume was as above, and concentrations were as follows: sgRNA1 (46.25 ng/μl), Cas9-protein (500 ng/μl), and 50 ng/μl of the myrGFP donor DNA. The donor DNA contained the *N*-myristoylation (myr) sequence, the GFP coding sequence, and the endopeptidase P2A coding sequence (table S1). Two nucleotides were introduced before the myr sequence to maintain the open reading frame*.* Gly-Ser-Gly coding linker was added between the myr/GFP and GFP/P2A sequences. The coding sequences were optimized for the codons used in the honey bee. Homologous (approximately 250 bp of the nucleotide sequences) were added to left and right to get target-specific insertion at the start codon of the *dsx* gene in exon 2 [National Center for Biotechnology Information (NCBI); gene ID: 725126; reference sequence: NC_037642; annotation: Amel_HAv3.1] (fig. S10 and table S1). This sequence was synthesized, cloned, and sequenced (standard gene, Eurofins, Ebersberg, Germany). The sequence was amplified using Phusion High-Fidelity DNA Polymerase (Thermo Fisher Scientific, Braunschweig, Germany) to generate the donor DNA for injection.

### DNA, RNA, cDNA, and PCR procedures

DNA was extracted using the innuPrep Mini Kit (Analytic Jena, Jena, Germany). RNA was isolated using TRIzol reagent as described (*42*). First-strand cDNA synthesis was performed using Oligo(dT)_18_ primer and the RevertAid reverse transcriptase kit following the instructions of the supplier (Thermo Fisher Scientific, Waltham, USA). Semiquantitative PCR amplifications were run under nonsaturating conditions and in technical triplicates for each bee sample using the housekeeping gene *elongation factor 1-alpha* (*Ef1*α; 5′-GATATCGCCCTGTGGAAGTTC-3′, 5′-GTAACATTCGCTCCAGCAGC-3′) as reference for quantitatively adjusting expression levels across different samples (*36*). Amplicons were resolved using standard agarose gel electrophoresis (*34*).

### Genotyping and sequencing and procedures

For genotyping, we used PCR standard procedures using Phusion High-Fidelity DNA Polymerase (Thermo Fisher Scientific, Waltham, USA). Bees with a *dsx^stop/stop^* genotype were identified in a two-step process. Frame shift mutation was preselected by length polymorphism using hexachlorofluorescein-labeled amplicons. The amplicons were run on the ABI 3130XL Genetic Analyzer, and length differences were identified using Peak Scanner software (Applied Biosystems). Length (base pair) analysis was performed using Peak Scanner software (Thermo Fisher Scientific). Bees with a possible frame shift were deep sequenced. At least 50,000 reads per amplicon and individual were generated using Illumina MiSeq machine (Illumina, San Diego, USA). The sequencing data were analyzed using the web-based galaxy platform (https://usegalaxy.org/) to characterize frame shift mutations and mosaicism. Unrelated sequences, which made up to 6% of the sequences, were removed. The queens carrying myrGFP coding sequence were in the first step identified by genotyping using DNA extracts. PCR_1 amplified the sequence of the upstream insertion site (5′-GATTCGTAATAATTCCTGTGC-3′, 5′-CTGCGATGCCAGAAGGATATGTG-3′; Eurofins, Ebersberg, Germany). PCR_2 amplified the sequence of the downstream insertion site (5′-CTGCGATGCCAGAAGGATATGTG-3′; 5′-GTCAAAGTAAGAGTAGCGGAAG-3′). PCR_3 amplified the wt sequence (5′-GATTCGTAATAATTCCTGTGC-3′; 5′-GTCAAAGTAAGAGTAGCGGAAG-3′). The sequences of the targeted insertion sites were deep sequenced using Illumina MiSeq machine following the procedure described above. To perform RNA-seq of the antennas, we pooled RNA extracts from five bees for each of three biological replicates and genotype. Library preparation was performed using 500 ng of RNA and the “VAHTS Universal RNA-seq Library Prep Kit for Illumina V6 with mRNA capture module version 7.0” (Vazyme Biotech co.). Bead-purified libraries were normalized and finally sequenced on the HiSeq 3000/4000 system (Illumina Inc.) with a read setup of SR 1 × 150 bp. The bcl2fastq2 conversion software (v2.20.0.422) was used to convert the bcl files to fastq files as well as for the adapter trimming and the demultiplexing. Approximately 4 to 12 million single-end reads with a length of 150 bp were mapped to the *A. mellifera* transcriptome (NCBI Assembly Amel_HAv3.1) using the kallisto software tool (https://pachterlab.github.io/kallisto/about.html). Estimated read counts were normalized using the transcripts-per-kilobase-million method. Differences in gene expression were calculated using DESeq2 (https://bioconductor.org/packages/release/bioc/html/DESeq2.html). Genes were differentially expressed (differentially expressed genes, DEGs) if adjusted *P* values for multiple testing (*P*_adj_) were < 0.05, and log_2_ fold change was greater than 1.5.

### Behavioral examination procedures

To start the group experiments, 1-day-old laboratory-reared mutated and wt worker bees together with 1-day-old wt worker bees reared in the hive were assembled together with a queen into a group of approximately 500 bees (table S19). All bees were tagged using unique 2D barcodes that enable single bee computer-based tracking with the Bee Behavioral Annotation System [BBAS; (*16*)]. The bee group was maintained under dark condition and room temperature on a comb, which provided honey and pollen ad libitum. At day 6, the group of bees was transferred to a brood comb with food, which mimics the condition of the nurse bees on a brood comb in a colony. We standardized these brood combs in the biological replicates. To generate a standard brood comb, we filled cells with pollen or sugar solution at specific locations of the comb (fig. S3 and table S5). For the two pollen areas, we distributed 30 g of pollen among cells (“Echter Deutscher Spezial Blütenpollen,” Werner-Seip-Biozentrum GmbH & Co. KG, Butzbach, Germany). The pollen in each cell was covered with 25 μl of sugar solution (70% w/v saccharose solution; “Ambrosia Futtersirup,” Nordzucker AG, Braunschweig, Germany). The two honey areas consisted of 550 cells in which 200 μl of the sugar solution per cell was added. The brood area consisted of a piece of comb harboring 151 larvae of the third to fourth instar stage, which was located in the center of the comb (fig. S3 and table S5). Bees on the standardized comb were kept in an incubator overnight at 34°C. When worker bees were 7 to 9 days old, computer-based tracking of the bees was performed in the dark at room temperature for 48 hours (*16*).

For each bee, the BBAS generated information about the position on the comb in the X/Y/Z orientation four times per second together with a video. Average detection rate of the bees was 0.80 (table S6). If the detection of a bee was less than 10% in an hour, this hour was excluded. If the number of excluding hours exceeds 11 hours in a day, the bee was excluded for that day. We obtained *n* = 47 *dsx^stop/stop^* and *n* = 49 wt reared worker bees in five group replicates with bees *n* ≥ 5 in each of the replicate (table S19). All the behavioral examinations of these worker bees were done randomized and blind, as the observer or the experimenter had no knowledge about the genotype of the bee under study. We computed the time the bees’ spent (min/hour) in the areas containing either food (pollen + honey) or larvae or areas that were empty. We computed the number of bees’ visits (visits/hour) in the area containing food or larvae. We computed walking distance (m/h). This information of the moving behavior was extracted from the tracking data of the first 24 hours using C^++^ and Java scripts. The bees’ trajectories were thereby not continuous. If only a single frame was missing in the trajectory, the gap was linearly interpolated. The single bee trajectories were kept separate. Sequences in the tracking data that possibly represent cell inspection, brood feeding, cleaning empty cells, food handling, antennation, begging, or trophallaxis behavior, we initially identified using machine-based trained “encounter” (*16*) and “head in cell” identifier; the latter we newly trained. Identifiers were implemented in the JAABA program (*16*, *77*). Cross-validation estimates from 10 cross-validation rounds (*16*) for our trained “head in cell” identifier showed that 91.8% of the frames were true positive, 87.3% true negative, 8.2% false positive, and 12.7% false negative. Subsequently, the bee behaviors were manually reanalyzed using the video records. We used VirtualDub software (VirtualDub-1.9.11, https://virtualdub.org/) and software addition (*78*) to label a single bee and this randomized and blinded in respect to the respective genotype, mutant versus wt laboratory-reared worker bees. We classified the encounter behavior by video observations from 60 min of tracking data into antennation, begging, or trophallaxis behavior (table S19). We classified the head into cell behavior from 150 min of tracking data (table S19) into cell inspection behavior if the time period was shorter than <5 s (*15*). For the head into cell behaviors with time periods larger or equal 5 s, we further classified the behavior into cleaning, food handling, and larval-feeding task behaviors on the basis of the cells the bees entered (*15*), which either contained food, larvae, or were empty.

### Petri dish behavioral examination procedures

Polystyrene petri dishes (14-cm diameter; VWR, International GmbH, Darmstadt, Germany) were used as arenas for sensorimotor behavioral examinations. We introduced eight ventilation holes in the side walls and three of these in the lid with a diameter size of 5 mm diameter. The arenas were placed on a paper with black lines forming grids of 1.5 cm^2^ (fig. S7A) (*79*). Each of the 10- to 13-days-old *dsx^stop/stop^* or wt laboratory-reared worker bees was tested in the arena together with two other wt worker bees from the tracking experiment. The experiment was run under red light condition under a laboratory hood. The worker bees were placed in the middle of the arena and were left 10 to 12 min before the assay started. Sensorimotor functions were tested using 100% isopropanol (our solvent control), the alarm pheromone component IPA (≥99%, water free, Sigma-Aldrich, Taufkirchen, Deutschland), or the repellent benzaldehyde (≥99%, Sigma-Aldrich, Taufkirchen Deutschland). The sequence of testing was as follows. A small strip of filter paper (75-mm diameter, grade 413; VWR, International GmbH, Darmstadt, Germany) was introduced for 1 min. Either 0.5 μl of isopropanol, IPA, or benzaldehyde was applied, which we led evaporate for 1 min before we introduced the filter paper for 1 min into the arena. We counted line crossing if the entire bee crossed a line of the grid. However, if a bee immediately turned around after crossing the line, we counted this event as a single crossing (*79*). Positive phototaxis was examined in the petri dish by counting whether or not the bee walked toward the light beam (*80*). Four LED light sources (220 lumen, 2700 K) were distributed around the arena (fig. S7B). Light pulse was given for 10 s using the light source with the largest distance toward the bee examined in the arena and then turned off for 10 s. This procedure was repeated six times. Arenas and the paper with the grid were replaced by a new one once another bee was examined. Video recordings of the behaviors were analyzed (60 fps, full HD, 44100 Hz; Casio Exilim Pro EX-F1) using VSDC Free Video Editor (Multilab LLC, https://videosoftdev.com/). The samples were randomized. The observer had no knowledge about the genotype of the bee.

### Morphological and anatomical examination procedures

For the morphological and anatomical examinations, the bees were anesthetized on ice. Body, appendages, and heads were examined under the stereo microscope. Head morphology was quantified by measuring head length and head width as marked in fig. S5. Pictures were taken using a binocular (S8 APO, Leica) with a camera (UI-1240LE-C-HQ) and the software uEye Cockpit (IDS). To dissect the brain, the head was fixed on wax plate and covered with ice-cold honeybee saline buffer [130 mM NaCl, 5 mM KCl, 4 mM MgCl_2_, 5 mM CaCl_2_, 15 mM Hepes, 25 mM glucose, and 150 mM sucrose (pH 7.2)] or with phosphate-buffered saline [(PBS) 145.3 mM NaCl, 8.4 mM Na_2_HPO_4_ × 2H_2_O, and 1.5 mM NaH_2_PO_4_ × H_2_O,(pH 7.4]. The head capsule was opened between the eyes to remove the brain. The brain was directly fixed in ice-cold 4% formaldehyde (v/v) in PBS (pH 7.4) at 4°C for a minimum of 24 hours. The brains were washed three times for 10 min in PBS, 10 min in PBS with Triton X-100 (2% PBS-T), and twice for 10 min in 0.2% PBS-T, followed by 1 to 2 hours of incubation in 0.2% PBS-T with 2% NGS (normal goat serum, Invitrogen, USA), which was done at room temperature on a shaker.

To obtain phalloidin staining (labeling f-actin) for anatomical examinations, fixed brains were incubated in 0.5% PBS-T with 5% NGS and 0.2 U of Alexa Flour 568 phalloidin (Molecular Probes, A-12380, Eugene, USA) for 2 days at 4°C. The brains were washed four times for 5 min in PBS and were dehydrated using an isopropanol series with PBS buffer (10, 30, 50, 70, and 90% and two times in 100% isopropanol) for 5 min on a shaker. The brains were cleared in methylsalycylate (MS; Sigma-Aldrich, Taufkirchen, Germany) and mounted in fresh MS solution. Malformations were reported when repeatedly observed in the same brain area.

To detect the GFP-labeled cells, we incubated the fixed brains in 0.2% PBS-T with 2% NGS and chicken anti-GFP (1:1000; Rockland Immunochemicals Inc., Limerick, PA, USA) for 4 days at 4°C. The brains were washed three times for 5 min in PBS. The brains were than treated with goat anti-chicken Alexa Fluor 488 (1:250; Thermo Fisher Scientific, Schwerte, Germany) and Alexa Flour 568 phalloidin in 0.2% PBS-T with 2% NGS for 2 days at 4°C. The brains were then washed three times for 5 min in PBS and dehydrated in an isopropanol series, cleared, and mounted in MS, as described above. All brains were stored at 4°C under dark condition until they were examined. Optical sections of the brain were generated with confocal laser scanning microscope (Leica TSC SP8 STED 3X, Leica Microsystems, Wetzlar, Germany) every 3.0 to 6.0 μm (*z*-stacks). Scans of brain were done with the 20× objective [multi/numerical aperture (NA) 0.75] using the Mosaic Merge function of the Leica Application Suite X 3.0.0 (LAS X, Leica Microsystems CMS, Wetzlar, Germany). We used the 40× objective (water/NA 1.10) for higher resolution scans. The *dsx^myrGFP/+^* worker progenies (*n* = 39) never showed the disruption of brain anatomy (Fig. 1C and movies S1 and S2). To quantify the size of structure or staining, we used FIJI (ImageJ 1.53c; Wayne Rasband, National Institutes of Health, USA), LAS X, and, Imaris software (Oxford Instruments, Abingdon, United Kingdom, version 9.1.2).

### Data analysis and statistics

Statistical analysis was performed using Systat (Systat Software GmbH, Erkrath, Germany) and IBM SPSS Statistics 27 software (IBM, Armonk, USA). MWU test was used for pairwise comparison. To test against zero (no change), one-sample Wilcoxon signed-rank test was used.
