# Supplementary material for: Dedicated developmental programing for group-supporting behaviors in eusocial honeybees
Source: Sci Adv. 2024 Nov 1;10(44):eadp3953. doi: 10.1126/sciadv.adp3953 (PMC11529710; doi:10.1126/sciadv.adp3953)
Supplement: Supplementary file 1 — Figs. S1 to S10 Tables S1 to S19 Legends for movies S1 to S23 [file sciadv.adp3953_sm.pdf]

Science Advances
AAAS

Supplementary Materials for

Dedicated developmental programing for group-supporting behaviors in eusocial honeybees

Vivien Sommer *et al.*

Corresponding author: Vivien Sommer, viviensommer1@gmail.com;
Martin Beye, martin.beye@uni-duesseldorf.de

Sci. Adv. **10**, eadp3953 (2024)
DOI: 10.1126/sciadv.adp3953

The PDF file includes:

Figs. S1 to S10
Tables S1 to S19
Legends for movies S1 to S23

Other Supplementary Material for this manuscript includes the following:

Movies S1 to S23

Supporting information Figures

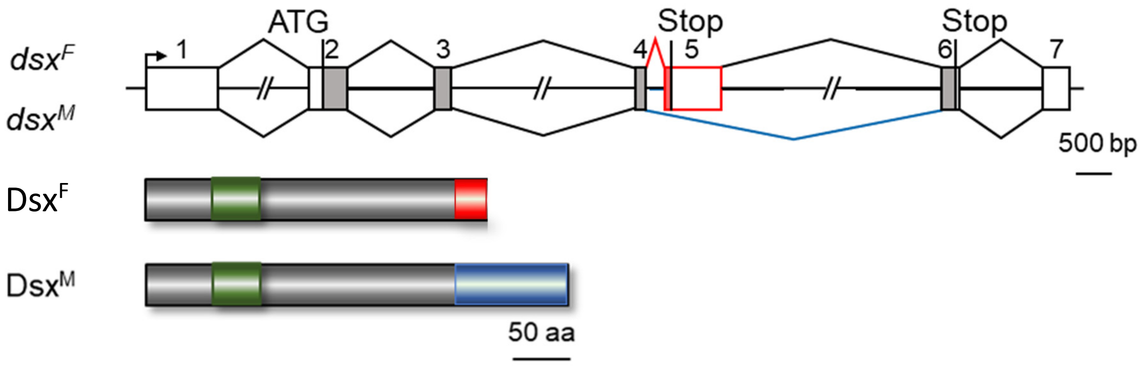

Figure S1. The sex-specific splicing scheme of the *dsx* gene in the honeybee. Above: boxes are the exons, and the interconnected lines indicate the splicing. Red lines/boxes indicate the female-specific inclusion of exon 5 via splice processing. The blue line indicates male-specific splicing and exclusion of female exon 5. Gray shaded boxes mark the ORF (open reading frame). Below: scheme of the protein structure highlighting the ZnF domain (DM domain; green) and the female- and male-specific peptide in red and blue, respectively. bp; base pairs. aa; amino acids.

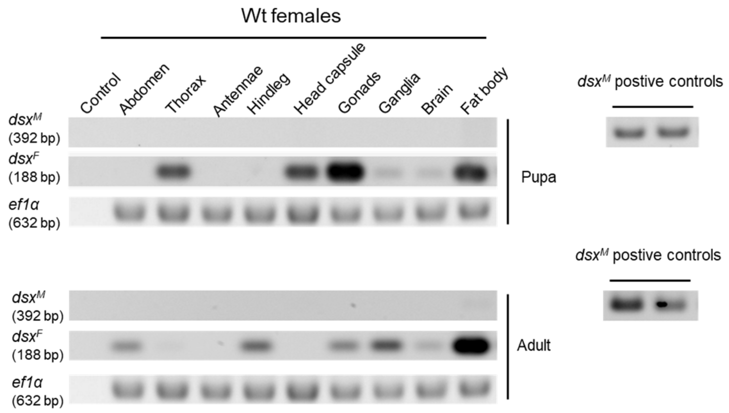

Figure S2: Sex-specific expression of *dsx* in worker bee tissues. Female (*dsx*^{F}) and male (*dsx*^{M}) *dsx* transcripts were examined in pupal stage 4 and 1-day-old adult worker bees. Amplicons from RT–PCRs were size-resolved and were semi-quantitatively adjusted across samples using *ef-1α (ef1α, elongation Factor 1α*) transcripts as a reference. Pictures are black and white inverted. Three biological each with three technical replicates were conducted: *ef1α*: *elongation Factor 1α*; Control: negative control for PCR. *dsx*^{M} positive controls: two cDNA samples were run as positive controls during *dsx*^{M} amplification experiments.

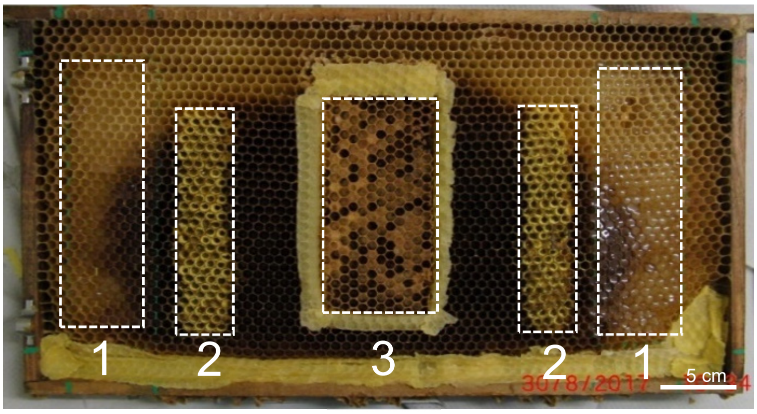

Figure S3. Brood comb to standardize workload for the worker bees in repeated tracking experiments. Sugar solution (1; mimicking honey), pollen (2) or larvae (3) were provided in the same areas across combs. The brood area consisted of 151 larvae at larval stages 3 and 4. 200 µl sugar solution per cell was provided. 30 g of ground pollen was split among the two pollen areas.

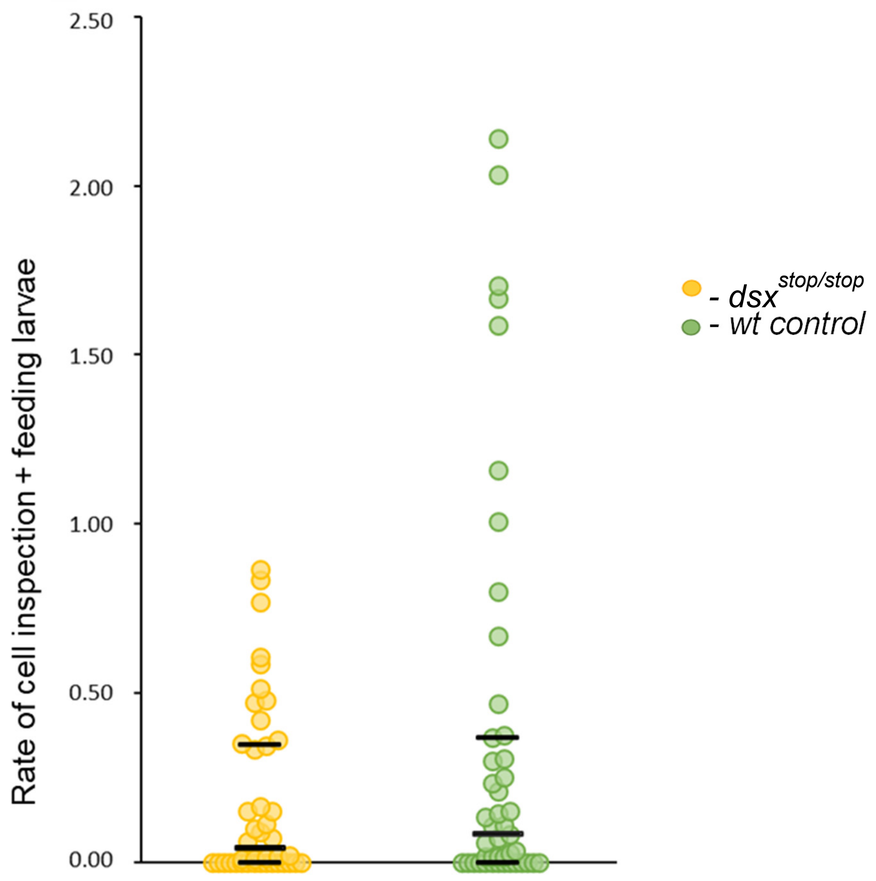

Figure S4. *dsx* gene activity is not required for the first entry into the larval cell. Cell inspection and larval feeding values are combined for this comparison (*P* = 0.365, z = 0.907; MWU test) (See Figure 5A and B).

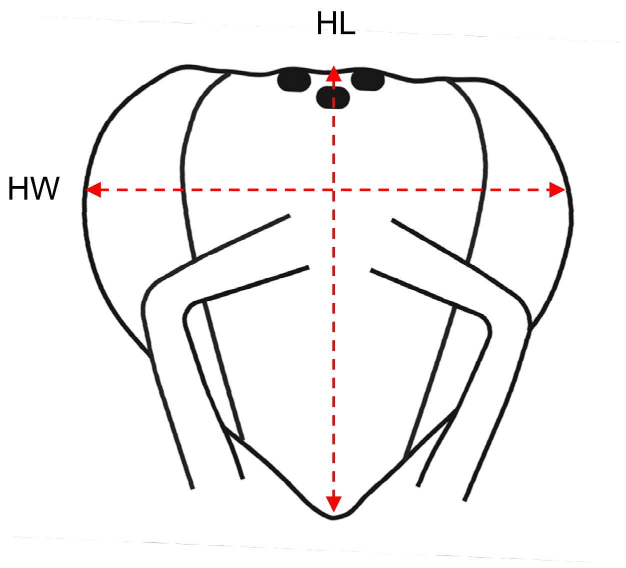

Figure S5. Measurements of honeybee head. Red lines represent the linear measurements of head length (HL) and width (HW).

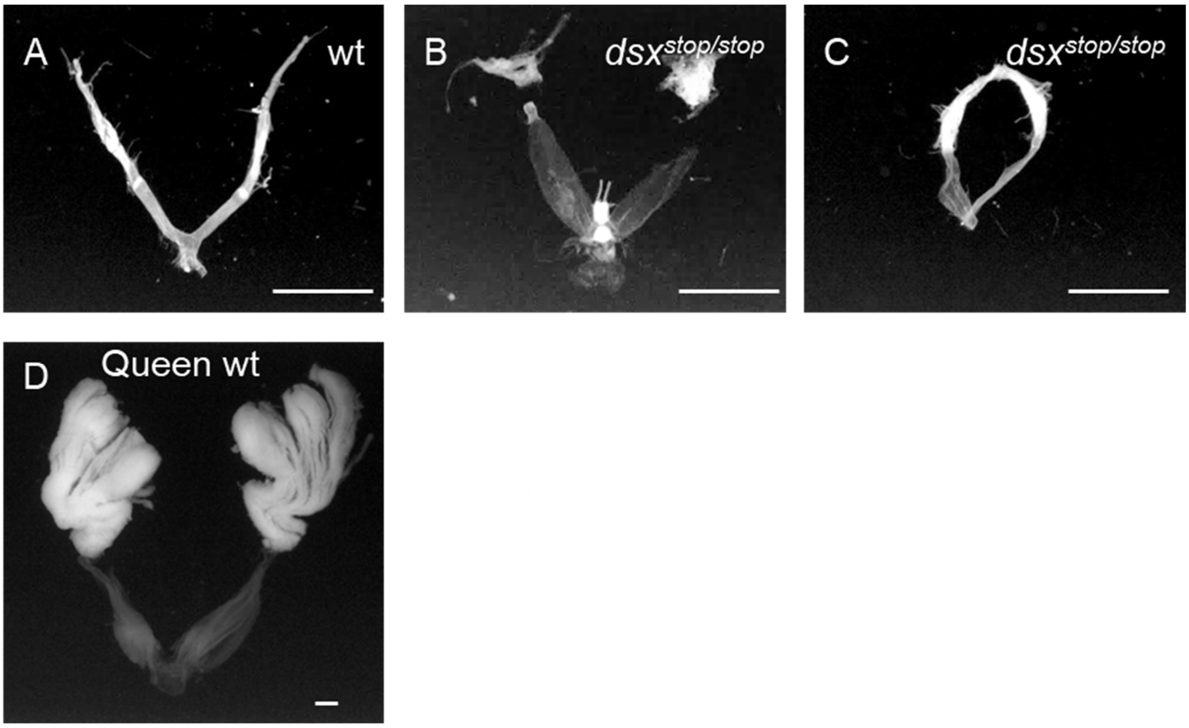

Figure S6. Worker ovaries from 10-13 days old worker bees. A. wt worker bee control **B.** *dsx*$^{stop/stop}$ worker bee with enlarged oviducts and intersex gonad. **C.** *dsx*$^{stop/stop}$ worker bee with reduced oviducts. **D.** Queen ovaries for comparison. Scale: 0.5 mm.

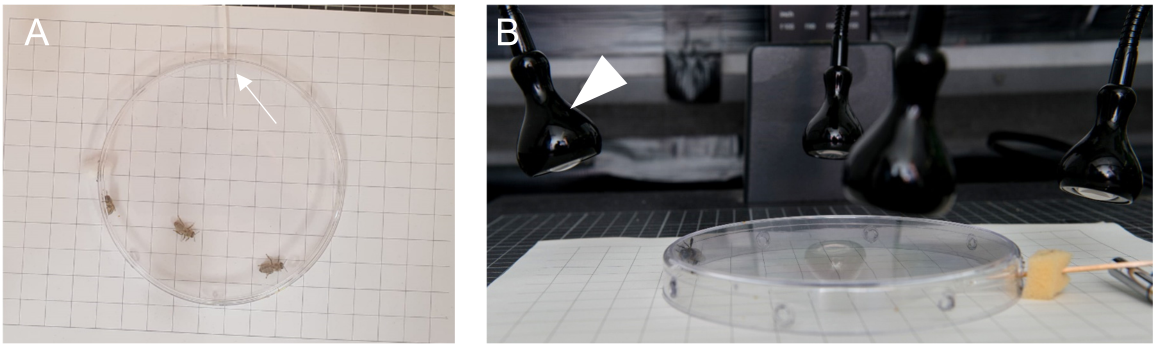

Figure S7. Petri dish behavioral assays for sensorimotor functions. Worker bees were examined in Petri dishes (14 cm). **A.** Example of *dsx*$^{stop/stop}$ worker bee odor sensorimotor examination in the presence of two hive-reared wt worker bees. Arrow shows the filter paper employed to provide the odor. **B.** Example of a wt worker bee examination of light responsiveness. The arrowhead marks the LED lamp.

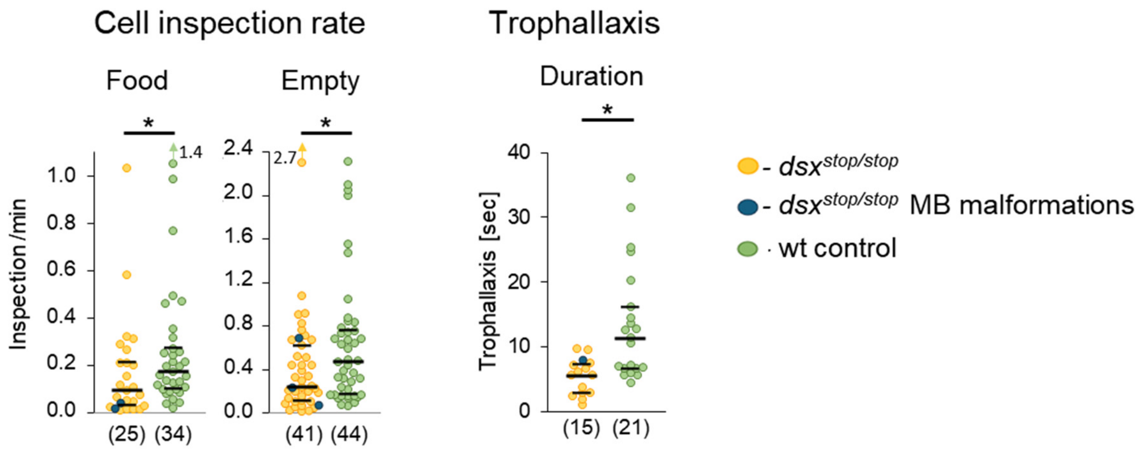

Figure S8. The dysfunctional task behaviors of mutant worker bees that have malformed MB brain structures. The median (middle line) and quartiles are presented. *n* values are shown in parentheses. min; minutes; sec; seconds; *; P < 0.05 (MWU test). Details see figure 5.

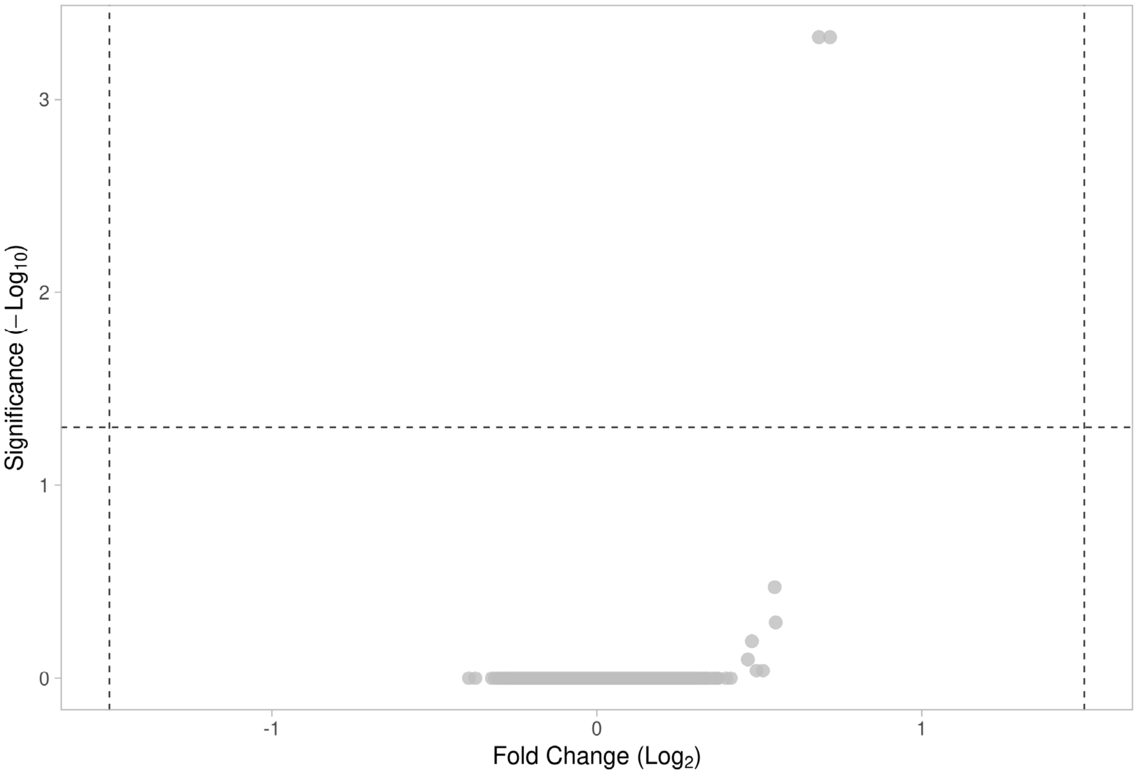

Figure S9. Differential gene expression in the antenna of *dsx*$^{stop/stop}$ versus wt worker bees. RNAseq was used, and 9361 genes were examined. Genes were differentially expressed (differentially expressed genes, DEGs) if adjusted P-values for multiple testing (Padj) were < 0.05 and log2 fold change was greater than 1.5 (dotted lines).

AACGAGGAATCGGGGGAAAGAAAACTGGTGTCGAAAATCGAATCTACGCCTCGACTACGTTTCGA
AACACGTGTTCTCGTTTTTTACAAGCGCGCGATAAAAGGATTAGAGAGAGAGAGAGAAAGGACAA
CGATAGAGGGACAAACAACCGTTCAAACATTTCATTGAGATTGTTCTTTGTAATTATGAAAAGGC
TGTGAATCGAGGTTACCTATGTATCGCGAAGAGAACGAGCAAAACAGAGCCGCGGACTTGGCTCC
CCAACAACCGAGTGGTGCAAACACGTTCGAGCGTTTGGAACATTCTCAGGATAGCAAAAATGGGG
ACGATGGTCCCAAGAAGGTGCAAACAGACGCTTCCTCTTCGACTAATACTCCAAAGCCGCGTGCA
CGGAATTGTGCACGATGTCTGAATCATCGGCTGGAGATCACCTTAAAATCGCACAAGAGGTACTG
CAAGTACCGTACTTGTACCTGCGAGAAGTGTAAGATCACTGCCAATCGGCAGCAAGTGATGCGGC
AGAATATGAAGCTGAAAAGACACCTGGCACAGGATAAAGTCAAAGTAAGAGTAGCGGAAGAG

Figure S10. The nucleotide sequence elements of *dsx* exon 2 were employed in this study. The nucleotide sequence of exon 2 is shown, and sequence elements highlighted; single guide RNAs sgRNA1(red box) and sgRNA2 (brownish box) target sequence and the sites of their possible double-strand break (underlined); start codon (green colored box); sequence encoding the DM DNA binding domain (light blue colored box); target sequence of oligonucleotide primers (grey boxes) used for genotyping.

Supporting information: Table

Table S1. Nucleotide sequences of the sgRNAs and myrGFP DNA fragment.

Name	Nucleotide sequence
dsx sgRNA1[1)]	**GAACGAGCAAAACAGAGCCG**GUUUUAGAGCUAGAAAUAGCAAGUUAAAAUAAGGCU<br>AGUCCGUUAUCAACUUGAAAAAGUGGCACCGAGUCGGUGCUUUU
dsx sgRNA2 [1)]	**GUGCACGAUGUCUGAAUCAU**GUUUUAGAGCUAGAAAUAGCAAGUUAAAAUAAGGCU<br>AGUCCGUUAUCAACUUGAAAAAGUGGCACCGAGUCGGUGCUUUU
myrGFP DNA fragment [2)]	GTTGCAGAACGAGGAATCGGGGGAAAGAAAACTGGTGTCGAAAATCGAATCTACGC<br>CTCGACTACGTTTCGAAACACGTGTTCTCGTTTTTTACAAGCGCGCGATAAAAGGA<br>TTAGAGAGAGAGAGAGAAAGGACAACGATAGAGGGACAAACAACCGTTCAAACATT<br>TCATTGAGATTGTTCTTTGTAATTATGAAAAGGCTGTGAATCGAGGTTACCTATGT<br>ATCGCGAAGAGAACGAGCAAAACAGAGGAATGGGCAATAAATGCTGCAGCAAAAGA<br>CAAGATCAAGAATTGGCTTTAGCGTATCCAACAGGAGGTTACAAGAAATCGGATTA<br>TACGTTCGGACAAACACATATCAATTCTAGCGGCGGTGGAAATATGGGTGGAGTGT<br>TGGGCCAAAAACATAACAATGGTGGATCGTTAGATTCTAGATATACGCCAGATCCT<br>AATCATAGAGGTCCATTGAAAATTGGAGGCAAAGGTGGAGTTGATATCATTAGACC<br>TAGAGGATCTATGAGCAAAGGAGAAGAACTGTTCACAGGTGTTGTGCCAATCTTAG<br>TTGAATTGGATGGCGATGTGAATGGACATAAATTCTCTGTGTCGGGTGAAGGAGAA<br>GGCGATGCTACGTATGGTAAATTGACATTAAAATTCATTTGCACTACGGGAAAACT<br>GCCAGTGCCTTGGCCAACATTGGTTACGACTTTAACATATGGTGTGCAATGCTTCA<br>GCAGATATCCTGATCATATGAAACAACATGATTTTTTCAAATCTGCGATGCCAGAA<br>GGATATGTGCAAGAAAGAACGATCTTTTTCAAAGATGATGGTAATTACAAAACAAG<br>AGCTGAAGTTAAATTCGAAGGAGATACGTTGGTGAATAGAATTGAATTAAAAGGTA<br>TCGATTTTAAAGAAGATGGAAATATTCTTGGTCATAAATTGGAATATAATTACAAC<br>AGCCATAATGTTTATATAATGGCTGATAAACAAAAAAATGGAATCAAAGTGAACTT<br>CAAAATTAGACATAATATAGAAGATGGTTCGGTTCAATTAGCGGATCATTACCAAC<br>AAAATACACCAATTGGAGATGGTCCTGTTCTGTTGCCAGATAATCATTATTTAAGC<br>ACGCAATCTGCTTTGTCGAAAGATCCAAATGAAAAAAGAGATCATATGGTGTTACT<br>TGAATTCGTTACAGCGGCTGGAATTACGCATGGTATGGATGAATTATATAAAGGAT<br>CTGGTGCTACAAATTTCTCTTTGTTAAAACAAGCGGGAGATGTGGAAGAAAATCCA<br>GGTCCTGCCGCGGACTTGGCTCCCCAACAACCGAGTGGTGCAAACACGTTCGAGCG<br>TTTGGAACATTCTCAGGATAGCAAAAATGGGGACGATGGTCCCAAGAAGGTGCAAA<br>CAGACGCTTCCTCTTCGACTAATACTCCAAAGCCGCGTGCACGGAATTGTGCACGA<br>TGTCTGAATCATCGGCTGGAGATCACCTTAAAATCGCACAAGAGGTACTGCAAGTA<br>CCGTACTTGTACCTGCGAGAAGTGTAAGATCA

1) bold letters indicate the target site in the genome.
2) *dsx* gene homologous sequence are shown in gray boxes, myristoylation (myr) coding sequence in yellow box, GFP coding sequence in green box, 2A peptide (P2A) coding sequence in blue box and the GS and GSG linkers in orange boxes. The first two start codons are underlined. To maintain the open reading frame, nucleotides were inserted (red letters). Codon usage were optimized for the honeybee.

Table S2. The rate of generating *dsx*$^{stop/stop}$ worker bees.

Genotype	# (%) of adult worker bees
dsx$^{stop/stop}$	67 (59)
dsx$^{nonstop/stop}$ (mono allelic stop)	26 (23)
Mosaic	15 (13)
Wildtype	7 (6)
Total	115

Table S3. **Nucleotide *dsx* exon 2 sequences (genotype) detected of the independently mutated and reared *dsx*$^{stop/stop}$ worker bees.** Yellow box: mediated early stop codon. aa: amino acids.

Wildtype

>allele a/b
ATGTATCGCGAAGAGAACGAGCAAAACAGAGCCGCGGACTTGGCTCCCCAACAACCGAGTGGTGCAAACACGT
TCGAGCGTTTGGAACATTCTCAGGATAGCAAAAATGGGGACGATGGTCCCAAGAAGGTGCAAACAGACGCTTC
CTCTTCGACTAATACTCCAAAGCCGCGTGCACGGAATTGTGCACGATGTCTGAATCATCGGCTGGAGATCACC
TTAAAATCGCACAAGAGGTACTGCAAGTACCGTACTTGTACCTGCGAGAAGTGTAAGATCACTGCCAATCGGC
AGCAAGTG

#13 i2-18

>allele a, aa 10
ATGTATCGCGAAGAGAACGAGCAAAACATGTGATCGGCTGGAGATCACCTTAAAATCGCACAAGAGGTACTGC
AAGTACCGTACTTGTACCTGCGAGAAGTGTAAGATCACTGCCAATCGGCAGCAAGTG

>allele b, aa 31
ATGTATCGCGAAGAGAACGAGCAAAACAGAGGACTTGGCTCCCCAACAACCGAGTGGTGCAAACACGTTCGAG
CGTTTGGAACATTCTCAGGATAGCAAAAATGGGGACGATGGTTCCAAGAAGGTGCAAACAGACGCTTCCTCTT
CGACTAATACTCCAAAGCCGCGTGCACGGAATTGTGCACGATGTCTGAATCTACGCCTCGGCTGGAGATCACC
TTAAAATCGCACAAGAGGTACTGCAAGTACCGTACTTGTACCTGCGAGAAGTGTAAGATCACTGCCAATCGGC
AGCAAGTG

#22 i2-18

>allele a, aa 66
ATGTATCGCGAAGAGAACGAGCAAAACAGAGGACTTGGCTCCCCAACAACCGAGTGGTGCAAACACGTTCGAG
CGTTTGGAACATTCTCAGGATAGCAAAAATGGGGACGATGGTTCCAAGAAGGTGCAAACAGACGCTTCCTCTT
CGACTAATACTCCAAAGCCGCGTGCACGGAATTGTGCACGATGTCTGAATCGTGATCATCGACTGGAGATCAC
CTTAAAATCGCACAAGAGGTACTGCAAGTACCGTACTTGTACCTGCGAGAAGTGTAAGATCACTGCCAATCGG
CAGCAAGTG

>allele b, aa 31
ATGTATCGCGAAGAGAACGAGCAAAACAGAGGACTTGGCTCCCCAACAACCGAGTGGTGCAAACACGTTCGAG
CGTTTGGAACATTCTCAGGATAGCAAAAATGGGGACGATGGTTCCAAGAAGGTGCAAACAGACGCTTCCTCTT
CGACTAATACTCCAAAGCCGCGTGCACGGAATTGTGCACGATGTCTGAATCGTGATCATCGACTGGAGATCAC
CTTAAAATCGCACAAGAGGTACTGCAAGTACCGTACTTGTACCTGCGAGAAGTGTAAGATCACTGCCAATCGG
CAGCAAGTG

#43 i2-18

>allele a, aa 32
ATGTATCGCGAAGAGAACGAGCAAAACAGACGCGGACTTGGCTCCCCAACAACCGAGTGGTGTAAACACGTTC
GAGCGTTTGGAACATTCTCAGGATAGCAAAAATGGGGACGATGGTTCCAAGAAGGTGCAAACAGACGCTTCCT
CTTCGACTAATACTCCAAAGCCGCGTGCACGGAATTGTGCACGATGTCTGAATCGTGCTGGAGATCACCTTAA
AATCGCACAAGAGGTACTGCAAGTACCGTACTTGTACCTGCGAGAAGTGTAAGATCACTGCCAATCGGCAGCA
AGTG

>allele b, aa 15
ATGTATCGCGAAGAGAACGAGCAAAACAGAGGCTGGAGATCACCTTAAAATCGCACAAGAGGTACTGCAAGTA
CCGTACTTGTACCTGCGAGAAGTGTAAGATCACTGCCAATCGGCAGCAAGTG

#58 i2-18

>allele a, aa 15
ATGTATCGCGAAGAGAACGAGCAAAACATCGGCTGGAGATCACCTTAAAATCGCACAAGAGGTACTGCAAGTA
CCGTACTTGTACCTGCGAGAAGTGTAAGATCACTGCCAATCGGCAGCAAGTG

>allele b, aa 15
ATGTATCGCGAAGAGAACGAGCAAAACAGAGGCTGGAGATCACCTTAAAATCGCACAAGAGGTACTGCAAGTA
CCGTACTTGTACCTGCGAGAAGTGTAAGATCACTGCCAATCGGCAGCAAGTG

#60 i2-18

>allele a, aa 35
ATGTATCGCGAAGAGAACGAGCAAAACAGATCTCCATCGGCTGGAGATCACCTTAAAATCGCACAAGAGGTAC
TGCAAGTACCGTACTTGTACCTGCGAGAAGTGTAAGATCACTGCCAATCGGCAGCAAGTG

>allele b, aa 15
ATGTATCGCGAAGAGAACGAGCAAAACAGAGGCTGGAGATCACCTTAAAATCGCACAAGAGGTACTGCAAGTA
CCGTACTTGTACCTGCGAGAAGTGTAAGATCACTGCCAATCGGCAGCAAGTG

#7 i3-18

>allele a, aa 15
ATGTATCGCGAAGAGAACGAGCAAAACATCGGCTGGAGATCACCTTAAAATCGCACAAGAGGTACTGCAAGTA
CCGTACTTGTACCTGCGAGAAGTGTAAGATCACTGCCAATCGGCAGCAAGTG

>allele b, aa 15
ATGTATCGCGAAGAGAACGAGCAAAACAGAGGCTGGAGATCACCTTAAAATCGCACAAGAGGTACTGCAAGTA
CCGTACTTGTACCTGCGAGAAGTGTAAGATCACTGCCAATCGGCAGCAAGTG

#29 i3-18

>allele a, aa 31
ATGTATCGCGAAGAGAACGAGCAAAACCGCGGACTTGGCTCCCCAACAACCGAGTGGTGCAAACACGTTCGAG
CGTTTGGAACATTCTCAGGATAGCAAAAATGGGGACGATGGTCCCAAGAAGGTGCAAACAGACGCTTCCTCTT
CGACTAATACTCCAAAGCCGCGTGCACGGAATTGTGCACGATGTCTGATGTCATCGGCTGGAGATCACCTTAA
AATCGCACAAGAGGTACTGCAAGTACCGTACTTGTACCTGCGAGAAGTGTAAGATCACTGCCAATCGGCAGCA
AGTG

>allele b, aa 31
ATGTATCGCGAAGAGAACGAGCAAAACAGAGGACTTGGCTCCCCAACAACCGAGTGGTGCAAACACGTTCGAG
CGTTTGGAACATTCTCAGGATAGCAAAAATGGGGACGATGGTTCCAAGAAGGTGCAAACAGACGCTTCCTCTT
CGACTAATACTCCAAAGCCGAGTGCACGGAATTGTGCACGATGTCTGAAGCATCGGCTGGAGATCACCTTAAA
ATCGCACAAGAGGTACTGCAAGTACCGTACTTGTACCTGCGAGAAGTGTAAGATCACTGCCAATCGGCAGCAA
GTG

#30 i3-18

>allele a, aa 30
ATGTATCGCGAAGAGAACGAGCAAAACAGACTTGGCTCCCCAACAACCGAGTGGTGCAAACACGTTCGAGCGT
TTGGAACATTCTCAGGATAGCAAAAATGGGGACGATGGTTCCAAGAAGGTGCAAACAGACGCTTCCTCTTCGA
CTAATACTCCAAAGCCGCGTGCACGGAATTGTGCACGATGTCGGCTGGAGATCACCTTAAAATCGCACAAGAG
GTACTGCAAGTACCGTACTTGTACCTGCGAGAAGTGTAAGATCACTGCCAATCGGCAGCAAGTG

>allele b, aa 15
ATGTATCGCGAAGAGAACGAGCAAAACAGAGGCTGGAGATCACCTTAAAATCGCACAAGAGGTACTGCAAGTA
CCGTACTTGTACCTGCGAGAAGTGTAAGATCACTGCCAATCGGCAGCAAGTG

#44 i3-18

>allele a, aa 31
ATGTATCGCGAAGAGAACGAGCAAAACAGAGGACTTGGCTCCCCAACAACCGAGTGGTGCAAACACGTTCGAG
CGTTTGGAACATTCTCAGGATAGCAAAAATGGGGACGATGGTTCCAAGAAGGTGCAAACAGACGCTTCCTCTT
CGACTAATACTCCAAAGCCGAGTGCACGGAATTGTGCACGATGTCTGAAGCATCGGCTGGAGATCACCTTAAA

ATCGCACAAGAGGTACTGCAAGTACCGTACTTGTACCTGCGAGAAGTGTAAGATCACTGCCAATCGGCAGCAA
GTG

>allele b, aa 36
ATGTATCGCGAAGAGAACGAGCAAAACAGAGGTTGGAGATCGGCTGGAGATCACCTTAAAATCGCACAAGAGG
TACTGCAAGTACCGTACTTGTACCTGCGAGAAGTGTAAGATCACTGCCAATCGGCAGCAAGTG

#51 i3-18

>allele a, aa 15
ATGTATCGCGAAGAGAACGAGCAAAACAGAGGCTGGAGATCACCTTAAAATCGCACAAGAGGTACTGCAAGTA
CCGTACTTGTACCTGCGAGAAGTGTAAGATCACTGCCAATCGGCAGCAAGTG

>allele b, aa 36
ATGTATCGCGAAGAGAACGAGCAAAACAGAGGTTGGAGATCGGCTGGAGATCACCTTAAAATCGCACAAGAGG
TACTGCAAGTACCGTACTTGTACCTGCGAGAAGTGTAAGATCACTGCCAATCGGCAGCAAGTG

#58 i3-18

>allele a, aa 19
ATGTATCGCGAAGAGAACGAGCAAAACAGAGCCGATGCCATCGGCTGGAGATCACCTTAAAATCGCACAAGAG
GTACTGCAAGTACCGTACTTGTACCTGCGAGAAGTGTAAGATCACTGCCAATCGGCAGCAAGTG

>allele b, aa 15
ATGTATCGCGAAGAGAACGAGCAAAACAGAGGCTGGAGATCACCTTAAAATCGCACAAGAGGTACTGCAAGTA
CCGTACTTGTACCTGCGAGAAGTGTAAGATCACTGCCAATCGGCAGCAAGTG

#69 i3-18

>allele a/b, aa 15
ATGTATCGCGAAGAGAACGAGCAAAACATCGGCTGGAGATCACCTTAAAATCGCACAAGAGGTACTGCAAGTA
CCGTACTTGTACCTGCGAGAAGTGTAAGATCACTGCCAATCGGCAGCAAGTG

#1 i4-18

>allele a, aa 31
ATGTATCGCGAAGAGAACGAGCAAAACCGCGGACTTGGCTCCCCAACAACCGAGTGGTGCAAACACGTTCGAG
CGTTTGGAACATTCTCAGGATAGCAAAAATGGGGACGATGGTTCCAAGAAGGTGCAAACAGACGCTTCCTCTT
CGACTAATACTCCAAAGCCGCGTGCACGGAATTGTGCACGATGTCTGAATCGGCTGGAGATCACCTTAAAATC
GCACAAGAGGTACTGCAAGTACCGTACTTGTACCTGCGAGAAGTGTAAGATCACTGCCAATCGGCAGCAAGTG

>allele b, aa 91
ATGTATCGCGAAGAGAACGAGCAAAACAGAGCGGACTTGGCTCCCCAACAACCGAGTGGTGCAAACACGTTCG
AGCGTTTGGAACATTCTCAGGATAGCAAAAATGGGGACGATGGTCCCAAGAAGGTGCAAACAGACGCTTCCTC
TTCGACTAATACTCCAAAGCCGCGTGCACGGAATTGTGCACGATGTCTGAATAATTCATCGGCTGGAGATCAC
CTTAAAATCGCACAAGAGGTACTGCAAGTACCGTACTTGTACCTGCGAGAAGTGTAAGATCACTGCCAATCGG
CAGCAAGTG

#33 i4-18

>allele a, aa 30
ATGTATCGCGAAGAGAACGAGCAAAACAGACTTGGCTCCCCAACAACCGAGTGGTGCAAACACGTTCGAGCGT
TTGGAACATTCTCAGGATAGCAAAAATGGGGACGATGGTTCCAAGAAGGTGCAAACAGACGCTTCCTCTTCGA
CTAATACTCCAAAGCCGCGTGCACGGAATTGTGCACGATGTCTGAAATCGGCTGGAGATCACCTTAAAATCGC
ACAAGAGGTACTGCAAGTACCGTACTTGTACCTGCGAGAAGTGTAAGATCACTGCCAATCGGCAGCAAGTG

>allele b, aa 15
ATGTATCGCGAAGAGAACGAGCAAAACAGAGGCTGGAGATCACCTTAAAATCGCACAAGAGGTACTGCAAGTA
CCGTACTTGTACCTGCGAGAAGTGTAAGATCACTGCCAATCGGCAGCAAGTG

#38 i4-18

>allele a, aa 72
ATGTATCGCGAAGAGAACGAGCAAAACAGAGCGGACTTGGCTCCCCAACAACCGAGTGGTGCAAACACGTTCG
AGCGTTTGGAACATTCTCAGGATAGCAAAAATGGGGACGATGGTTCCAAGAAGGTGCAAACAGACGCTTCCTC
TTCGACTAATACTCCAAAGCCGCGTGCACGGAATTGTGCACGATGTCTGAATCACGGCTGGAGATCACCTTAA
AATCGCACAAGAGGTACTGCAAGTACCGTACTTGTACCTGCGAGAAGTGTAAGATCACTGCCAATCGGCAGCA
AGTG

>allele b, aa 15
ATGTATCGCGAAGAGAACGAGCAAAACATCGGCTGGAGATCACCTTAAAATCGCACAAGAGGTACTGCAAGTA
CCGTACTTGTACCTGCGAGAAGTGTAAGATCACTGCCAATCGGCAGCAAGTG

#42 i4-18

>allele a/b, aa 64
ATGTATCGCGAAGAGAACGAGCAAAACAGAGCGGACTGGCTCCCCAACAACCGAGTGGTGCAAACACGTTCGA
GCGTTTGGAACATTCTCAGGATAGCAAAAATGGGGACGATGGTTCCAAGAAGGTGCAAACAGACGCTTCCTCT
TCGACTAATACTCCAAAGCCGCGTGCACGGAATTGTGCACGATGTCTGAATCGGCTGGAGATCACCTTAAAAT
CGCACAAGAGGTACTGCAAGTACCGTACTTGTACCTGCGAGAAGTGTAAGATCACTGCCATCGGCAGCAAGTG

#49 i4-18

>allele a, aa 31
ATGTATCGCGAAGAGAACGAGCAAAACAGAGGACTTGGCTCCCCAACAACCGAGTGGTGCAAACACGTTCGAG
CGTTTGGAACATTCTCAGGATAGCAAAAATGGGGACGATGGTTCCAAGAAGGTGCAAACAGACGCTTCCTCTT
CGACTAATACTCCAAAGCCGAGTGCACGGAATTGTGCACGATGTCTGAAGCATCGGCTGGAGATCACCTTAAA
ATCGCACAAGAGGTACTGCAAGTACCGTACTTGTACCTGCGAGAAGTGTAAGATCACTGCCAATCGGCAGCAA
GTG

>allele b, aa 15
ATGTATCGCGAAGAGAACGAGCAAACAATCGGCTGGAGATCACCTTAAAATCGCACAAGAGGTACTGCAAGTA
CCGTACTTGTACCTGCGAGAAGTGTAAGATCACTGCCAATCGGCAGCAAGTG

#52 i4-18

>allele a, aa 15
ATGTATCGCGAAGAGAACGAGCAAAACATCGGCTGGAGATCACCTTAAAATCGCACAAGAGGTACTGCAAGTA
CCGTACTTGTACCTGCGAGAAGTGTAAGATCACTGCCAATCGGCAGCAAGTG

>allele b, aa 67 deletion of ZnF domain
ATGTATCGCGAAGAGAACGAGCAAAACAGTC[- 168bp]CAGATCGGCTGGAGATCACCTTAAAATCGCACA
AGAGGTACTGCAAGTACCGTACTTGTACCTGCGAGAAGTGTAAGATCACTGCCAATCGGCAGCAAGTG

#53 i4-18

>allele a, aa 15
ATGTATCGCGAAGAGAACGAGCAAAACAGAGGCTGGAGATCACCTTAAAATCGCACAAGAGGTACTGCAAGTA
CCGTACTTGTACCTGCGAGAAGTGTAAGATCACTGCCAATCGGCAGCAAGTG

>allele b, aa 17
ATGTATCGCGAAGAGAACGAGCAAAACAGAGCCATCGGCTGGAGATCACCTTAAAATCGCACAAGAGGTACTG
CAAGTACCGTACTTGTACCTGCGAGAAGTGTAAGATCACTGCCAATCGGCAGCAAGTG

#66 i4-18

>allele a, aa 15
ATGTATCGCGAAGAGAACGAGCAAAACAGAGGCTGGAGATCACCTTAAAATCGCACAAGAGGTACTGCAAGTA
CCGTACTTGTACCTGCGAGAAGTGTAAGATCACTGCCAATCGGCAGCAAGTG

>allele b, aa 35
ATGTATCGCGAAGAGAACGAGCAAAACAGATTCAGATCGGCTGGAGATCACCTTAAAATCGCACAAGAGGTAC
TGCAAGTACCGTACTTGTACCTGCGAGAAGTGTAAGATCACTGCCAATCGGCAGCAAGTG

#17 i1-19

>allele a, aa 19
ATGTATCGCGAAGAGAACGAGCAAAACGTTGTGGAGATCATCGGCTGGAGATCACCTTAAAATCGCACAAGAG
GTACTGCAAGTACCGTACTTGTACCTGCGAGAAGTGTAAGATCACTGCCAATCGGCAGCAAGTG

>allele b, aa 91
ATGTATCGCGAAGAGAACGAGCAAAACAGAGCGGACTTGGCTCCCCAACAACCGAGTGGTGCAAACACGTTCG
AGCGTTTGGAACATTCTCAGGATAGCAAAAATGGGGACGATGGTTCCAAGAAGGTGCAAACAGACGCTTCCTC
TTCGACTAATACTCCAAAGCCGCGTGCACGGAATTGTGCACGATGTCTGAATCCATCATCGGCTGGAGATCAC
CTTAAAATCGCACAAGAGGTACTGCAAGTACCGTACTTGTACCTGCGAGAAGTGTAAGATCACTGCCAATCGG
CAGCAAGTG

#43 i1-19

>allele a, aa 35
ATGTATCGCGAAGAGAACGAGCAAAACAGAGGTCGATCGGCTGGAGATCACCTTAAAATCGCACAAGAGGTAC
TGCAAGTACCGTACTTGTACCTGCGAGAAGTGTAAGATCACTGCCAATCGGCAGCAAGTG

>allele b, aa 34
ATGTATCGCGAAGAGAACGAGCAAAACAGAGCATCGGCTGGAGATCACCTTAAAATCGCACAAGAGGTACTGC
AAGTACCGTACTTGTACCTGCGAGAAGTGTAAGATCACTGCCAATCGGCAGCAAGTG

#1 i2-19

>allele a, aa 33
ATGTATCGCGAAGAGAACGAGCAAAACAGATCGGCTGGAGATCACCTTAAAATCGCACAAGAGGTACTGCAAG
TACCGTACTTGTACCTGCGAGAAGTGTAAGATCACTGCCAATCGGCAGCAAGTG

>allele b, aa 31
ATGTATCGCGAAGAGAACGAGCAAAACAGAGGACTTGGCTCCCCAACAACCGAGTGGTGCAAACACGTTCGAG
CGTTTGGAACATTCTCAGGATAGCAAAAATGGGGACGATGGTCCCAAGAAGGTGCAAACAGACGCTTCCTCTT
CGACTAATACTCCAAAGCCGCGTGCACGGAATTGTGCACGATGTCTGAATCGGCTGGAGATCACCTTAAAATC
GCACAAGAGGTACTGCAAGTACCGTACTTGTACCTGCGAGAAGTGTAAGATCACTGCCAATCGGCAGCAAGTG

#7 i2-19

>allele a, aa 31
ATGTATCGCGAAGAGAACGAGCAAAACAGAGGACTTGGCTCCCCAACAACCGAGTGGTGCAAACACGTTCGAG
CGTTTGGAACATTCTCAGGATAGCAAAAATGGGGACGATGGTTCCAAGAAGGTGCAAACAGACGCTTCCTCTT
CGACTAATACTCCAAAGCCGCGTGCACGGAATTGTGCACGATGTCTGAATCGGCTGGAGATCACCTTAAAATC
GCACAAGAGGTACTGCAAGTACCGTACTTGTACCTGCGAGAAGTGTAAGATCACTGCCAATCGGCAGCAAGTG

>allele b, aa 90
ATGTATCGCGAAGAGAACGAGCAAAACAGAGCGGACTTGGCTCCCCAACAACCGAGTGGTGCAAACACGTTCG
AGCGTTTGGAACATTCTCAGGATAGCAAAAATGGGGACGATGGTCCCAAGAAGGTGCAAACAGACGCTTCCTC

TTCGACTAATACTCCAAAGCCGCGTGCACGGAATTGTGCACGATGTCTGAATTCATCGGCTGGAGATCACCTT
AAAATCGCACAAGAGGTACTGCAAGTACCGTACTTGTACCTGCGAGAAGTGTAAGATCACTGCCAATCGGCAG
CAAGTG

#8 i2-19

>allele a, aa 15
ATGTATCGCGAAGAGAACGAGCAAAACATCGGCTGGAGATCACCTTAAAATCGCACAAGAGGTACTGCAAGTA
CCGTACTTGTACCTGCGAGAAGTGTAAGATCACTGCCAATCGGCAGCAAGTG

>allele b, aa 20
ATGTATCGCGAAGAGAACGAGCAAAACAGAGCTCGGCTCGTGATCGGCTGGAGATCACCTTAAAATCGCACAA
GAGGTACTGCAAGTACCGTACTTGTACCTGCGAGAAGTGTAAGATCACTGCCAATCGGCAGCAAGTG

#9 i2-19

>allele a, aa 17
ATGTATCGCGAAGAGAACGAGCAAAACAGATCCATCGGCTGGAGATCACCTTAAAATCGCACAAGAGGTACTG
CAAGTACCGTACTTGTACCTGCGAGAAGTGTAAGATCACTGCCAATCGGCAGCAAGTG

>allele b, aa 19
ATGTATCGCGAAGAGAACGAGCAAAACAGAGATCCAGCCATCGGCTGGAGATCACCTTAAAATCGCACAAGAG
GTACTGCAAGTACCGTACTTGTACCTGCGAGAAGTGTAAGATCACTGCCAATCGGCAGCAAGTG

#13 i2-19

>allele a, aa 15
ATGTATCGCGAAGAGAACGAGCAAAACAGAGGCTGGAGATCACCTTAAAATCGCACAAGAGGTACTGCAAGTA
CCGTACTTGTACCTGCGAGAAGTGTAAGATCACTGCCAATCGGCAGCAAGTG

>allele b, aa 51
ATGTATCGCGAAGAGAACGAGCAAAACAGTTCGAGCGTTTGGAACATTCTCAGGATAGCAAAAATGGGGACGA
TGGTCCCAAGAAGGTGCAAACAGACGCTTCCTCTTCGACTAATACTCCAAAGCCGCGTGCACGGAATTGTGCA
CGATGTCTGAATCGGCTGGAGATCACCTTAAAATCGCACAAGAGGTACTGCAAGTACCGTACTTGTACCTGCG
AGAAGTGTAAGATCACTGCCAATCGGCAGCAAGTG

#14 i2-19

>allele a/b, aa 15
ATGTATCGCGAAGAGAACGAGCAAAACATCGGCTGGAGATCACCTTAAAATCGCACAAGAGGTACTGCAAGTA
CCGTACTTGTACCTGCGAGAAGTGTAAGATCACTGCCAATCGGCAGCAAGTG

#18 i2-19

>allele a, aa 10
ATGTATCGCGAAGAGAACGAGCAAAACAGATAACGGACTAGCCTTATTTTAGCGGATTGGCTCCCCAACAACC
GAGTGGTGCAAACACGTTCGAGCGTTTGGAACATTCTCAGGATAGCAAAAATGGGGACGATGGTCCCAAGAAG
GTGCAAACAGACGCTTCCTCTTCGACTAATACTCCAAAGCCGCGTGCACGGAATTGTGCACGATGTCTGAATC
GTGCTGGAGATCACCTTAAAATCGCACAAGAGGTACTGCAAGTACCGTACTTGTACCTGCGAGAAGTGTAAGA
TCACTGCCAATCGGCAGCAAGTG

>allele b, aa 31

ATGTATCGCGAAGAGAACGAGCAAAACAGAGGACTTGGCTCCCCAACAACCGAGTGGTGCAAACACGTTCGAG
CGTTTGGAACATTCTCAGGATAGCAAAAATGGGGACGATGGTCCCAAGAAGGTGCAAACAGACGCTTCCTCTT
CGACTAATACTCCAAAGCCGCGTGCACGGAATTGTGCACGATGTCTGAATCATCGTCACATCGGCTGGAGATC
ACCTTAAAATCGCACAAGAGGTACTGCAAGTACCGTACTTGTACCCGCGAGAAGTGTAAGATCACTGCCAATC
GGCAGCAAGTG

#19 i2-19

>allele a/b, aa 28
ATGTATCGCGAAGACAACGATAGAGGGACAAACAACCGTTCAAACATTTCATTGAGATTGTTCTTTGTAATTA
TGAAAAGGCTGTGAATCGAGGTTACCTATGTATCGCGAAGAGAACGAGCAAAACAGAGCAAAGCACCGACTCG
GTGCCACTTTTTCAAGTTGATAACGGACTAGCCTTATTTTCTCCATCGGCTGGAGATCACCTTAAAATCGCAC
AAGAGGTACTGCAAGTACCGTACTTGTACCTGCGAGAAGTGTAAGATCACTGCCAATCGGCAGCAAGTGGAGA
ACGAGCAAAACATCGGCTGGAGATCACCTTAAAATCGCACAAGAGGTACTGCAAGTACCGTACTTGTACCTGC
GAGAAGTGTAAGATCACTGCCAATCGGCAGCAAGTG

#22 i2-19

>allele a, aa 62
ATGTATCGCGAAGAGAACGAGCAAAACAGAGGGCTCCCCAACAACCGAGTGGTGCAAACACGTTCGAGCGTTT
GGAACATTCTCAGGATAGCAAAAATGGGGACGATGGTCCCAAGAAGGTGCAAACAGACGCTTCCTCTTCGACT
AATACTCCAAAGCCGCGTGCACGGAATTGTGCACGATGTCTGAATCGGCTGGAGATCACCTTAAAATCGCACA
AGAGGTACTGCAAGTACCGTACTTGTACCTGCGAGAAGTGTAAGATCACTGCCAATCGGCAGCAAGTGC

>allele b, aa 31
ATGTATCGCGAAGAGAACGAGCAAAACAGAGGACTTGGCTCCCCAACAACCGAGTGGTGCAAACACGTTCGAG
CGTTTGGAACATTCTCAGGATAGCAAAAATGGGGACGATGGTCCCAAGAAGGTGCAAACAGACGCTTCCTCTT
CGACTAATACTCCAAAGCCGCGTGCACGGAATTGTGCACGATGTCTGAATCGGCTGGAGATCACCTTAAAATC
GCACAAGAGGTACTGCAAGTACCGTACTTGTACCTGCGAGAAGTGTAAGATCACTGCCAATCGGCAGCAAGTG
G

#23 i2-19

>allele a, aa 21
ATGTATCGCGAAGAGAACGAGCAAAACAGAGACGGAACGATGAACATCGGCTGGAGATCACCTTAAAATCGCA
CAAGAGGTACTGCAAGTACCGTACTTGTACCTGCGAGAAGTGTAAGATCACTGCCAATCGGCAGCAAGTG

>allele b, aa 64
ATGTATCGCGAAGAGAACGAGCAAAACAGAGGGACTTGGCTCCCCAACAACCGAGTGGTGCAAACACGTTCGA
GCGTTTGGAACATTCTCAGGATAGCAAAAATGGGGACGATGGTTCCAAGAAGGTGCAAACAGACGCTTCCTCT
TCGACTAATACTCCAAAGCCGCGTGCACGGAATTGTGCACGATGTCTGAATCGGCTGGAGATCACCTTAAAAT
CGCACAAGAGGTACTGCAAGTACCGTACTTGTACCTGCGAGAAGTGTAAGATCACTGCCAATAGGCAGCAAGT
GC

#31 i2-19

>allele a, aa 15
ATGTATCGCGAAGAGAACGAGCAAAACATCGGCTGGAGATCACCTTAAAATCGCACAAGAGGTACTGCAAGTA
CCGTACTTGTACCTGCGAGAAGTGTAAGATCACTGCCAATCGGCAGCAAGTG

>allele b, aa 15
ATGTATCGCGAAGAGAACGAGCAAAACAGAGGCTGGAGATCACCTTAAAATCGCACAAGAGGTACTGCAAGTA
CCGTACTTGTACCTGCGAGAAGTGTAAGATCACTGCCAATCGGCAGCAAGTG

#33 i2-19

>allele a, aa 15
ATGTATCGCGAAGAGAACGAGCAAAACATCGGCTGGAGATCACCTTAAAATCGCACAAGAGGTACTGCAAGTA
CCGTACTTGTACCTGCGAGAAGTGTAAGATCACTGCCAATCGGCAGCAAGTG

>allele b, aa 31
ATGTATCGCGAAGAGAACGAGCAAAACAGAGGACTTGGCTCCCCAACAACCGAGTGGTGCAAACACGTTCGAG
CGTTTGGAACATTCTCAGGATAGCAAAAATGGGGACGATGGTCCCAAGAAGGTGCAAACAGACGCTTCCTCTT
CGACTAATACTCCAAAGCCGCGTGCACGGAATTGTGCACGATGTCTGAATCTGCAAGATACTGCAAGTACCGT
ACTTGTACCTGCGAGAAGTGTAAGATCACTGCCAATCGGCAGCAAGTG

#37 i2-19

>allele a, aa 15
ATGTATCGCGAAGAGAACGAGCAAAACATCGGCTGGAGATCACCTTAAAATCGCACAAGAGGTACTGCAAGTA
CCGTACTTGTACCTGCGAGAAGTGTAAGATCACTGCCAATCGGCAGCAAGTG

>allele b, aa 15
ATGTATCGCGAAGAGAACGAGCAAAACAGAGGCTGGAGATCACCTTAAAATCGCACAAGAGGTACTGCAAGTA
CCGTACTTGTACCTGCGAGAAGTGTAAGATCACTGCCAATCGGCAGCAAGTG

#38 i2-19

>allele a, aa 19
ATGTATCGCGAAGAGAACGAGCAAAACAGAGGATCTCCGATCGGCTGGAGATCACCTTAAAATCGCACAAGAG
GTACTGCAAGTACCGTACTTGTACCTGCGAGAAGTGTAAGATCACTGCCAATCGGCAGCAAGTG

>allele b, aa 31
ATGTATCGCGAAGAGAACGAGCAAAACCGCGGACTTGGCTCCCCAACAACCGAGTGGTGCAAACACGTTCGAG
CGTTTGGAACATTCTCAGGATAGCAAAAATGGGGACGATGGTCCCAAGAAGGTGCAAACAGACGCTTCCTCTT
CGACTAATACTCCAAAGCCGCGTGCACGGAATTGTGCACGATGTCTGAATCGGCTGGAGATCACCTTAAAATC
GCACAAGAGGTACTGCAAGTACCGTACTTGTACCTGCGAGAAGTGTAAGATCACTGCCAATCGGCAGCAAGTG
C

#42 i2-19

>allele a, aa 15
ATGTATCGCGAAGAGAACGAGCAAAACAGAGGCTGGAGATCACCTTAAAATCGCACAAGAGGTACTGCAAGTA
CCGTACTTGTACCTGCGAGAAGTGTAAGATCACTGCCAATCGGCAGCAAGTG

>allele b, aa 31
ATGTATCGCGAAGAGAACGAGCAAAACAGAGGACTTGGGTCCCCAACAACCGAGTGGTGCAAACACGTTCGAG
CGTTTGGAACATTCTCAGGATAGCAAAAATGGGGACGATGGTCCCAAGAAGGTGCAAACAGACGCTTCCTCTT
CGACTAATACTCCAAAGCCGCGTGCACGGAATTGTGCACGATGTCTGAATCATTAATCATCGGCTGGAGATCA
CCTTAAAATCGCACAAGAGGTACTGCAAGTACCGTACTTGTACCTGCGAGAAGTGTAAGATCACTGCCAATCG
GCAGCAAGTG

#40 i2-19

>allele a, aa 32

ATGTATCGCGAAGAGAACGAGCAAAACAGACTTGGACTTGGCTCCCCAACAACCGAGTGGTGCAAACACGTTC
GAGCGTTTGGAACATTCTCAGGATAGCAAAAATGGGGACGATGGTCCCAAGAAGGTGCAAACAGACGCTTCCT
CTTCGACTAATACTCCAAAGCCGCGTGCACGGAATTGTGCACGATGTCTGAATTTTCGCGATCGGCTGGAGAT
CACCTTAAAATCGCACAAGAGGTACTGCAAGTACCGTACTTGTACCTGCGAGAAGTGTAAGATCACTGCCAAT
CGGCAGCAAGTG

>allele b, aa 67
ATGTATCGCGAAGAGAACGAGCAAAACAGAGACTTGGCTTGGACTTGGCTCCCCAACAACCGAGTGGTGCAAA
CACGTTCGAGCGTTTGGAACATTCTCAGGATAGCAAAAATGGGGACGATGGTCCCAAGAAGGTGCAAACAGAC
GCTTCCTCTTCGACTAATACTCCAAAGCCGCGTGCACGGAATTGTGCACGATGTCTGAATCGTGGCTGGAGGA
GTCCATCGGCTGGAGATCACCTTAAAATCGCACAAGAGGTACTGCAAGTACCGTACTTGTACCTGCGAGAAGT
GTAAGATCACTGCCAATCGGCAGCAAGTG

#50 i2-19

>allele a, aa 15
ATGTATCGCGAAGAGAACGAGCAAAACAGAGGCTGGAGATCACCTTAAAATCGCACAAGAGGTACTGCAAGTA
CCGTACTTGTACCTGCGAGAAGTGTAAGATCACTGCCAATCGGCAGCAAGTG

>allele b, aa 31
ATGTATCGCGAAGAGAACGAGCAAAACCGCGGACTTGGCTCCCCAACAACCGAGTGGTGCAAACACGTTCGAG
CGTTTGGAACATTCTCAGGATAGCAAAAATGGGGACGATGGTTCCAAGAAGGTGCAAACAGACGCTTCCTCTT
CGACTAATACTCCAAAGCCGCGTGCACGGAATTGTGCACGATGTCTGAATCGGCTGGAGATCACCTTAAAATC
GCACAAGAGGTACTGCAAGTACCGTACTTGTACCTGCGAGAAGTGTAAGATCACTGCCAATCGGCAGCAAGTG

#176 i2-19

>allele a, aa 22
ATGTATCGCGAAGAGAACGAGCAAAACAGAGACAGGATCGGCTCCAGCATCGGCTGGAGATCACCTTAAAATC
GCACAAGAGGTACTGCAAGTACCGTACTTGTACCTGCGAGAAGTGTAAGATCACTGCCAATCGGCAGCAAGTG

>allele b, aa 15
ATGTATCGCGAAGAGAACGAGCAAAACAGATCGGCTGGAGATCACCTTAAAATCGCACAAGAGGTACTGCAAG
TACCGTACTTGTACCTGCGAGAAGTGTAAGATCACTGCCAATCGGCAGCAAGTG

#1 i3-19

>allele a, aa 15
ATGTATCGCGAAGAGAACGAGCAAAACAGAGGCTGGAGATCACCTTAAAATCGCACAAGAGGTACTGCAAGTA
CCGTACTTGTACCTGCGAGAAGTGTAAGATCACTGCCAATCGGCAGCAAGTG

>allele b, aa 17
ATGTATCGCGAAGAGAACGAGCAAAACAGAGAAATCGGCTGGAGATCACCTTAAAATCGCACAAGAGGTACTG
CAAGTACCGTACTTGTACCTGCGAGAAGTGTAAGATCACTGCCAATCGGCAGCAAGTG

#2 i3-19

>allele a, aa 15
ATGTATCGCGAAGAGAACGAGCAAAACATCGGCTGGAGATCACCTTAAAATCGCACAAGAGGTACTGCAAGTA
CCGTACTTGTACCTGCGAGAAGTGTAAGATCACTGCCAATCGGCAGCAAGTG

>allele b, aa 19
ATGTATCGCGAAGAGAACGAGCAAAACAGAGAAACCGAGATCGGCTGGAGATCACCTTAAAATCGCACAAGAG
GTACTGCAAGTACCGTACTTGTACCTGCGAGAAGTGTAAGATCACTGACAATCGGCAGCAAGTG

#10 i3-19

>allele a, aa 15
ATGTATCGCGAAGAGAACGAGCAAAACATCGGCTGGAGATCACCTTAAAATCGCACAAGAGGTACTGCAAGTA
CCGTACTTGTACCTGCGAGAAGTGTAAGATCACTGCCAATGGCAGCAAGTG

>allele b, aa 31
ATGTATCGCGAAGAGAACGAGCAAAACAGAGGACTTGGCTCCCCAACAACCGAGTGGTGCAAACACGTTCGAG
CGTTTGGAACATTCTCAGGATAGCAAAAATGGGGACGATGGTCCCAAGAAGGTGCAAACAGACGCTTCCTCTT
CGACTAATACTCCAAAGCCGCGTGCACGGAATTGTGCACGATGTCTGCATCGGCTGGAGATCACCTTAAAATC
GCACAAGAGGTACTGCAAGTACCGTACTTGTACCTGCGAGAAGTGTAAGATCACTGCCAATCGGCAGCAAGTG

#17 i3-19

>allele a, aa 15
ATGTATCGCGAAGAGAACGAGCAAAACATCGGCTGGAGATCACCTTAAAATCGCACAAGAGGTACTGCAAGTA
CCGTACTTGTACCTGCGAGAAGTGTAAGATCACTGCCAATCGGCAGCAAGTG

>allele b, aa 31
ATGTATCGCGAAGAGAACGAGCAAAACAGAGGACTTGGCTCCCCAACAACCGAGTGGTGCAAACACGTTCGAG
CGTTTGGAACATTCTCAGGATAGCAAAAATGGGGACGATGGTTCCAAGAAGGTGCAAACAGACGCTTCCTCTC
CGACTAATACTCCAAAGCCGCGTGCACGGAATTGTGCACGATGTCTGAATCGGCTGGAGATCACCTTAAAATC
GCACAAGAGGTACTGCAAGTACCGTACTTGTACCTGCGAGAAGTGTAAGATCACTGCCAATCGGCAGCAAGTG

#19 i3-19

>allele a, aa 15
ATGTATCGCGAAGAGAACGAGCAAAACATCGGCTGGAGATCACCTTAAAATCGCACAAGAGGTACTGCAAGTA
CCGTACTTGTACCTGCGAGAAGTGTAAGATCACTGCCAATCGGCAGCAAGTG

>allele b, aa 15
ATGTATCGCGAAGAGAACGAGCAAAACAGCGGCTGGAGATCACCTTAAAATCGCACAAGAGGTACTGCAAGTA
CCGTACTTGTACCTGCGAGAAGTGTAAGATCACTGCCAATCGGCAGCAAGTG

#20 i3-19

>allele a, aa 33
ATGTATCGCGAAGAGAACGAGCAAAACAGATCGGCTGGAGATCACCTTAAAATCGCACAAGAGGTACTGCAAG
TACCGTACTTGTACCTGCGAGAAGTGTAAGATCACTGCCAATCGGCAGCAAGTG

>allele b, aa 31
ATGTATCGCGAAGAGAACGACCAAAACCGCGGACTTGGCTCCCCAACAACCGAGTGGTGCAAACACGTTCGAG
CGTTTGGAACATTCTCAGGATAGCAAAAATGGGGACGATGGTCCCAAGAAGGTGCAAACAGACGCTTCCTCTT
CGACTAATACTCCAAAGCCGCGTGCACGGAATTGTGCACGATGTCTGAATCGGCTGGAGATCACCTTAAAATC
GCACAAGAGGTACTGCAAGTACCGTACTTGTACCTGCGAGAAGTGTAAGATCACTGCCAATCGGCAGCAAGTG

#26 i3-19

>allele a, aa 15
ATGTATCGCGAAGAGAACGAGCAAAACATCGGCTGGAGATCACCTTAAAATCGCACAAGAGGTACTGCAAGTA
CCGTACTTGTACCTGCGAGAAGTGTAAGATCACTGCCAATCGGCAGCAAGTG

>allele b, aa 33
ATGTATCGCGAAGAGAACGAGCAAAACAGATCGGCTGGAGATCACCTTAAAATCGCACAAGAGGTACTGCAAG
TACCGTACTTGTACCTGCGAGAAGTGTAAGATCACTGCCAATCGGCAGCAAGTG

#27 i3-19

>allele a, aa 15
ATGTATCGCGAAGAGAACGAGCAAAACATCGGCTGGAGATCACCTTAAAATCGCACAAGAGGTACTGCAAGTA
CCGTACTTGTACCTGCGAGAAGTGTAAGATCACTGCCAATCGGCAGCAAGTG

>allele b, aa 30
ATGTATCGCGAAGAGAACGAGCAAAACAGACTTGGCTCCCCAACAACCGAGTGGTGCAAACACGTTCGAGCGT
TTGGAACATTCTCAGGATAGCAAAAATGGGGACGATGGTCCCAAGAAGGTGCAAACAGACGCTTCCTCTTCGA
CTAATACTCCAAAGCCGCGTGCACGGAATTGTGCACGATGTCTGAATCGGCTGGAGATCACCTTAAAATCGCA
CAAGAGGTACTGCAAGTACCGTACTTGTACCTGCGAGAAGTGTAAGATCACTGCCAATCGGCAGCAAGTG

#41 i3-19

>allele a, aa 15
ATGTATCGCGAAGAGAACGAGCAAAACATCGGCTGGAGATCACCTTAAAATCGCACAAGAGGTACTGCAAGTA
CCGTACTTGTACCTGCGAGAAGTGTAAGATCACTGCCAATCGGCAGCAAGTG

>allele b, aa 19
ATGTATCGCGAAGAGAACGAGCAAAACAGAGAACAAGACATCGGCTGGAGATCACCTTAAAATCGCACAAGAG
GTACTGCAAGTACCGTACTTGTACCTGCGAGAAGTGTAAGATCACTGCCAATCGGCAGCAAGTG

Table S4. Survival of the laboratory reared experimental worker bees.

Treatment	# of 2-day old larvae	# of eclosed adults	% survival to adult stage	Fisher´s exact test, *P*-value, df = 1
Injected	732	171	23.1 %	0.25
Control	907	235	25.9 %	

Table S5. Sizes of the standardized areas on the combs for each replicate.

SD = standard deviation.

replicate	brood (cm^2)	pollen (cm^2)	Sugar solution (cm^2)
i2-2018	56.72	32.33	74.31
i3-2018	46.50	29.70	76.49
i4-2018	75.39	34.09	74.81
i2-2019	69.34	27.90	71.12
i3-2019	61.12	32.44	72.00
mean	61.81	31.29	73.75
SD +/-	10.02	2.20	1.95

Table S6. Detection rate of the bees.

SD = standard deviation.

Replicate	% detection rate	Maximal # of bees detected
i2-2018	0.79	465
i3-2018	0.70	461
i4-2018	0.75	460
i2-2019	0.91	461
i3-2019	0.83	447
mean	0.80	459
SD +/-	0.07	6.1

Table S7. The rearing of the 151 larvae in each replicate.

SD = standard deviation.

Replicate	% of larvae
i2-2018	53.6
i3-2018	68.2
i4-2018	70.2
i2-2019	63.6
i3-2019	58.3
mean	62.8
SD +/-	6.2

Table S8. The rate of cell inspection behaviors for *dsx*$^{stop/stop}$ and wildtype (wt) worker bees.

SD = standard deviation. MWU = Mann-Whitney U-test. min: minutes

Cell inspection /min	*dsx*$^{stop/stop}$				wt control				MWU *P*-value	z
	n	Median	Mean	± SD	*n*	Median	Mean	± SD		
All	42	0.49	0.63	± 0.53	45	0.87	1.05	± 0.85	0.006	2.74
Larvae	26	0.15	0.20	± 0.18	31	0.15	0.35	± 0.46	0.67	0.83
Food	25	0.09	0.18	± 0.23	33	0.17	0.27	± 0.29	0.04	2.04
Empty	41	0.24	0.41	± 0.47	44	0.48	0.63	± 0.59	0.04	2.03

Table S9. The rate of task behaviors.

SD = standard deviation. MWU = Mann-Whitney U-test. min: minutes

**Task behavior /min**	***dsx*** $^{stop/stop}$				**wt control**				MWU *P*-value	z
	n	Median	Mean	± SD	*n*	Median	Mean	± SD		
All tasks	39	0.17	0.27	± 0.27	39	0.39	0.42	± 0.34	0.02	2.35
Larval feeding	20	0.09	0.13	± 0.11	22	0.21	0.25	± 0.21	0.054	1.93
Food handling	15	0.03	0.05	± 0.03	21	0.04	0.05	± 0.03	0.53	0.65
Cleaning empty cells	33	0.15	0.22	± 0.24	37	0.18	0.27	± 0.27	0.29	1.05

Table S10. The duration of the task behaviors.

SD = standard deviation. MWU = Mann-Whitney U-test. sec: seconds

Duration task behavior [sec]	*dsx*$^{stop/stop}$				wt control				MWU *P*-value	z
	n	Median	Mean	± SD	*n*	Median	Mean	± SD		
All	39	13.42	16.17	± 7.67	39	17.84	20.27	± 10.05	0.04	2.03
Larval feeding	20	16.69	21.12	± 12.99	22	16.57	19.72	± 11.12	0.82	0.23
Food handling	15	6.75	17.37	± 22.22	21	13.50	25.81	± 29.19	0.02	2.28
Cleaning empty cells	33	13.42	15.03	± 7.33	37	15.37	22.76	± 21.63	0.21	1.25

Table S11. Rate of trophallaxis related behaviors.

SD = standard deviation. MWU = Mann-Whitney U-test. min: minutes

Rate /min	***dsx***$^{stop/stop}$				**wt control**				MWU *P*-value	z
	n	Median	Mean	± SD	*n*	Median	Mean	± SD		
Antennation	42	0.39	0.43	± 0.31	47	0.38	0.50	± 0.38	0.65	0.52
Begging	25	0.11	0.11	± 0.09	34	0.07	0.12	± 0.18	0.71	0.67
Trophallaxis	15	0.07	0.12	± 0.09	21	0.09	0.14	± 0.07	0.95	0.06

Table S12. Duration of the trophallaxis behavior.

SD = standard deviation. MWU = Mann-Whitney U-test. sec: seconds

**Duration [sec]**	***dsx***$^{stop/stop}$				**wt control**				MWU *P*-value	z
	n	Median	Mean	± SD	*n*	Median	Mean	± SD		
Food exchange	15	5.50	5.2	± 2.83	21	11.25	13.49	± 9.11	0.001	3.23

Table S13. Walking distance of the *dsx*$^{stop/stop}$ and the wildtype (wt) worker bees on the comb.

SD = standard deviation. MWU = Mann-Whitney U-test. m: meter; h: hour

**Walking behavior [m/h]**	***dsx*$^{stop/stop}$ (*n* = 47)**			**wt control (*n* = 49)**			MWU *P*-value	z
	Median	Mean	± SD	Median	Mean	± SD		
Distance	0.59	0.64	± 0.25	0.53	0.62	± 0.33	0.21	1.26

Table S14. The number of visits the experimental bees make to the different areas of the comb.
SD = standard deviation. MWU = Mann-Whitney U-test.

Visits per hour [visits/h]	*dsx*$^{stop/stop}$ (*n* = 47)			wt control (*n* = 49)			MWU *P*-value	z
	Median	Mean	± SD	Median	Mean	± SD		
Brood	1.25	1.31	± 0.61	1.22	1.42	± 0.50	0.85	0.19
Food	2.96	3.77	± 3.10	3.21	4.88	± 2.34	0.17	1.36

Table S15. The time the *dsx*$^{stop/stop}$ and wildtype (wt) worker bees spent in the different areas of the comb.

SD = standard deviation. MWU = Mann-Whitney U-test.

**Time spent in area [min/h]**	***dsx*$^{stop/stop}$ (*n* = 47)**			**wt control (*n* = 49)**			MWU *P*-value	z
	Median	Mean	± SD	Median	Mean	± SD		
Larvae	2.48	3.22	± 3.19	1.85	2.61	±1.75	0.33	0.97
Food	4.32	5.22	± 5.55	5.99	5.98	± 3.51	0.06	1.86
Empty	16.79	17.52	± 7.06	18.31	18.17	± 4.50	0.36	0.92

Table S16. The morphology and anatomy of the *dsx*$^{stop/stop}$ and wildtype (wt) worker bees.

Structure	# of *dsx*$^{stop/stop}$ with malformed structure (# examined)	# of wt worker bees with malformed structure (# examined)	Fisher's exact test *P*-value df = 1
Head	0 (29)	0 (26)	1
Body morphology	0 (17)	0 (11)	1
Abdominal segments [1)]	0 (17)	0 (11)	1
Antennal segments [2)]	0 (24)	0 (26)	1
Ovaries	6 (12)	0 (5)	0.10
Hypopharyngeal gland	0 (23)	0 (27)	1

[1)] wt worker bees have female-specific 6 abdominal segments.
[2)] wt worker bees have female-specific 13 antennal segments.

Table S17. The number of *dsx*$^{stop/stop}$ worker bees with maformations in the brain.

Group	Malformation	No malformation	Fisher's exact test, *P* value, df=1
dsx$^{stop/stop}$	6	20	0.008
Wildtype control	0	29	

Table S18. The location of malformations in specific brain structures of the *dsx*$^{stop/stop}$ worker bees.

*(A: extra structure, A^M: multiple such structures, B: deformed structure, C: misplaced structure, l-CA: lateral Calyx, m-CA: medial Calyx, LH: lateral horn)

			Affected structure *						Diameter of additional structure [µm]					
Bee ID	Replicate	Genotype allele 1/ allele 2	left l-CA	left m-CA	right m-CA	right l-CA	left LH	right LH	left l-CA	left m-CA	right m-CA	right l-CA	right LH	left LH
17	i1-19	stop/stop			A^M, B	A^M, B	A				27.3 to 40.3	21.8 to 71.5	47.6	
7	i2-19	stop/stop			A	A					23.9	21.6		
9	i2-19	stop/stop				A						41.6		
31	i2-19	stop/stop					A						24.5	
19	i3-19	stop/stop	A						20.0					
69	i3-18	stop/stop	A	A	C	A			40.0	33.3		46.6		

Table S19. Number of the *dsx*$^{stop/stop}$, the wt laboratory and the hive reared worker bees in the different behavioral analyses and biological replicates.

Replicate	# of bees				
	Type	Assembled 1)	Moving 2)	Encounters3)	Cell inspection/task4)
I2 2018	*dsx*$^{stop/stop}$	8	5	5	5
	wt lab	50	8	8	6
	Wildtype	444	-	-	-
I3 2018	*dsx*$^{stop/stop}$	14	7	5	6
	wt lab	42	8	6	7
	Wildtype	439	-	-	-
I4 2018	*dsx*$^{stop/stop}$	10	10	8	7
	wt lab	18	8	8	7
	Wildtype	474	-	-	-
I2 2019	*dsx*$^{stop/stop}$	19	16	15	15
	wt lab	57	16	16	13
	Wildtype	417	-	-	-
I3 2019	*dsx*$^{stop/stop}$	12	9	9	9
	wt lab	36	9	9	12
	Wildtype	443	-	-	-

1 Number at the begin of the tracking.
2 Number detected and computer-based examined for 24 hours
3 Number detected and computer-based annotated in the time frame examined (antennation, begging and trophallaxis behavior)
4 Number detected and computer-based annotated in the time frame examined (cell inspection/task behaviors).

Supporting information: Movies

Movie S1.

A middle brain z-stack of a wt worker bee which is stained with phalloidin.

Movie S2.

A middle brain z-stack of a *dsx* ^{myrGFP} worker bee which is stained with phalloidin and anti-GFP. Only the phalloidin staining is shown.

Movie S3.

A middle brain z-stack of a *dsx* ^{myrGFP} worker bee which is stained with phalloidin and anti-GFP. Only the anti-GFP staining is shown.

Movie S4.

A middle brain z-stack of a *dsx* ^{myrGFP} queen bee which is stained with phalloidin and anti-GFP. Only the phalloidin staining is shown.

Movie S5.

A middle brain z-stack of a *dsx* ^{myrGFP} queen bee which is stained with Phalloidin and anti-GFP. Only the anti-GFP staining is shown.

Movie S6.

Example of a laboratory reared *dsx*$^{stop/stop}$ worker bee (highlighted with the ID # 60) showing cell inspection behavior. The head is in the cell and the antennae are not visible. Movie is at half speed.

Movie S7.

Example of a laboratory reared wt worker bee (highlighted with the ID # 99) showing two cell inspection behaviors. The head is in the cell and the antennae are not visible. Movie is at half speed.

Movie S8.

Example of a laboratory reared *dsx*$^{stop/stop}$ worker (highlighted with the ID # 13) bee showing task behavior (≥ 5 sec head in cell). The head is in the cell and the antennae are not visible. Movie is at half speed.

Movie S9.

Example of a laboratory reared wt worker bee (highlighted with the ID # 63) showing task behavior (≥ 5 sec head in cell). The head is in the cell and the antennae are not visible.

Movie S10.

Example of a colony reared wt worker bee showing antennation behavior (marked by a circle). The bee encounters another bee. They face each other and have repeated contact with their antennae.

Movie S11.

Example of a colony reared wt worker bee showing begging behavior (marked by a circle). The bee encounters another bee. The bee to the left display begging behavior. It moves the head toward the other bee and reaches with its forelegs the other bee.

Movie S12.

Example of a colony reared wt worker bee showing trophallaxis behavior. The bee has contact via its proboscis with another worker bee, which indicates food sharing. Occasionally, one bee contacts the other bee with its forelegs and antennae during this behavior.

Movie S13.

Example of a laboratory reared $dsx^{stop/stop}$ worker bee (ID # 176) showing antennation behavior with another wt worker bee (ID # 479). They face each other and have repeated contact with their antennae. Movie is at half speed.

Movie S14.

Example of wt a laboratory reared worker bee (ID # 78) showing antennation behavior with another wt worker bee (ID # 146). They face each other and have repeated contact with their antennae. Movie is at half speed.

Movie S15.

Example of a laboratory reared $dsx^{stop/stop}$ worker bee (ID # 29) showing begging behavior. Bees have repeated contacts with their antennae to other wt bees (ID # 160). The respective bee moves its head towards the other bee and touches it with its front legs. Movie is at half speed.

Movie S16.

Example of a laboratory reared wt worker bee (ID # 93) showing begging behavior. Bees have repeated contacts with their antennae to other wt bees (ID # 434). The respective bee moves its head towards the other bee and touches it with its front legs. Movie is at half speed.

Movie S17.

Example of a laboratory reared *dsx*$^{stop/stop}$ worker bee (ID # 42) showing food sharing behavior (trophallaxis). The bees have contact via its expanded proboscis. Occasionally, the one bee contacts the other bee with its forelegs during this behavior. Movie is at half speed.

Movie S18.

Example of a laboratory reared wt worker bee (ID # 63) showing food sharing behavior (trophallaxis). The bees have contact via its expanded proboscis. Occasionally, the one bee contacts the other bee with its forelegs during this behavior during this behavior. Movie is at half speed.

Movie S19.

The middle brain z-stack of *dsx*$^{stop/stop}$ worker bee ID #17. Multiple additional structures (marked by arrows) and deformed lip (marked by a circle) in the medial and lateral right calyx. Additional structure (marked by a circle) in the lateral left horn.

Movie S20.

The middle brain z-stack of *dsx*$^{stop/stop}$ worker bee ID #7. Additional structures (marked by arrows) in the medial and lateral left calyx.

Movie S21.

The middle brain z-stack of *dsx*$^{stop/stop}$ worker bee ID #9. Additional structure (marked by an arrow) in the lateral calyx.

Movie S22.

The middle brain z-stack of *dsx*$^{stop/stop}$ worker bee ID #31. Additional structure in the lateral left horn area.

Movie S23.

The middle brain z-stack of *dsx*$^{stop/stop}$ worker bee ID #19. Additional structures (marked by arrows) in the lateral and medial left calyx.
